# Autophagy Is an Innate Mechanism Associated with Leprosy Polarization

**DOI:** 10.1371/journal.ppat.1006103

**Published:** 2017-01-05

**Authors:** Bruno Jorge de Andrade Silva, Mayara Garcia de Mattos Barbosa, Priscila Ribeiro Andrade, Helen Ferreira, José Augusto da Costa Nery, Suzana Côrte-Real, Gilberto Marcelo Sperandio da Silva, Patricia Sammarco Rosa, Mario Fabri, Euzenir Nunes Sarno, Roberta Olmo Pinheiro

**Affiliations:** 1 Leprosy Laboratory; Oswaldo Cruz Institute; Oswaldo Cruz Foundation, FIOCRUZ; Rio de Janeiro, Brazil; 2 Structural Biology Laboratory; Oswaldo Cruz Institute; Oswaldo Cruz Foundation, FIOCRUZ; Rio de Janeiro, Brazil; 3 Evandro Chagas National Institute of Infectology; FIOCRUZ; Rio de Janeiro, Brazil; 4 Laboratory Animal House; Lauro de Souza Lima Institute; São Paulo, Brazil; 5 Department of Dermatology; University of Cologne; Cologne, Germany; 6 Center for Molecular Medicine; University of Cologne; Cologne, Germany; New Jersey Medical School, UNITED STATES

## Abstract

Leprosy is a chronic infectious disease that may present different clinical forms according to the immune response of the host. Levels of IFN-γ are significantly raised in paucibacillary tuberculoid (T-lep) when compared with multibacillary lepromatous (L-lep) patients. IFN-γ primes macrophages for inflammatory activation and induces the autophagy antimicrobial mechanism. The involvement of autophagy in the immune response against *Mycobacterium leprae* remains unexplored. Here, we demonstrated by different autophagic assays that LC3-positive autophagosomes were predominantly observed in T-lep when compared with L-lep lesions and skin-derived macrophages. Accumulation of the autophagic receptors SQSTM1/p62 and NBR1, expression of lysosomal antimicrobial peptides and colocalization analysis of autolysosomes revealed an impairment of the autophagic flux in L-lep cells, which was restored by IFN-γ or rapamycin treatment. Autophagy PCR array gene-expression analysis revealed a significantly upregulation of autophagy genes (*BECN1*, *GPSM3*, *ATG14*, *APOL1*, and *TPR*) in T-lep cells. Furthermore, an upregulation of autophagy genes (*TPR*, *GFI1B* and *GNAI3*) as well as LC3 levels was observed in cells of L-lep patients that developed type 1 reaction (T1R) episodes, an acute inflammatory condition associated with increased IFN-γ levels. Finally, we observed increased BCL2 expression in L-lep cells that could be responsible for the blockage of BECN1-mediated autophagy. In addition, *in vitro* studies demonstrated that dead, but not live *M*. *leprae* can induce autophagy in primary and lineage human monocytes, and that live mycobacteria can reduce the autophagy activation triggered by dead mycobacteria, suggesting that *M*. *leprae* may hamper the autophagic machinery as an immune escape mechanism. Together, these results indicate that autophagy is an important innate mechanism associated with the *M*. *leprae* control in skin macrophages.

## Author Summary

Leprosy is an interesting model to study immune responses in humans due to the dichotomy observed among the poles of the disease. While in the self-limited tuberculoid form (T-lep) there are high systemic levels of the cytokine IFN-γ, in the clinically progressive lepromatous form (L-lep) low IFN-γ levels are found. IFN-γ activates an antimicrobial mechanism called autophagy, which has been implicated in control of *Mycobacterium tuberculosis* infection. However, the role played by autophagy in the immunopathogenesis of leprosy remains unknown. Here we show that autophagy was differentially regulated in T-lep and L-lep patients. In T-lep skin lesion cells autophagy contributes for bacilli control, whereas in L-lep cells the BCL2-mediated block of autophagy promotes the mycobacterial persistence. We also observed that IFN-γ may counteract the inhibition of autophagy triggered by *M*. *leprae* infection in L-lep macrophages. In addition, the levels of autophagy were restored in L-lep patients who developed the reversal reaction, an inflammatory state associated with augmented IFN-γ, which is the most important cause of nerve damage and deformities in leprosy. These findings suggest that the modulation of autophagy has the potential to be useful in the treatment of the disease, and provides new insights to prevent leprosy reactional episodes.

## Introduction

Leprosy is a chronic infectious disease that is gradually decreasing in many countries; however rates of new case detection remain at almost the same level globally and in different regions [1]. It is caused by *Mycobacterium leprae*, an intracellular pathogen that preferentially infects macrophages (MΦs) and Schwann cells [2]. Ridley and Jopling described a spectrum in which clinical manifestations correlate with the type of immune response [3]. At one end of the spectrum, tuberculoid leprosy (T-lep) is characterized by a strong cellular immune response to the pathogen, few skin lesions presenting well-formed granulomas and well-differentiated type 1 MΦs, and only rare mycobacteria. At the opposite end of the spectrum, lepromatous leprosy (L-lep) patients have a disseminated form, with several skin lesions, absence of granuloma formation, high amounts of type 2 foamy MΦs loaded with bacilli, and anti-*M*. *leprae* antibodies [2–4]. The existence of both poles is a direct reflection of the cytokine patterns found in their respective skin lesions. Whereas T-lep patients show a strong Th1 profile, including IFN-γ, TNF, and IL-15, L-lep patients have a predominantly Th2 type response, typical of IL-4, IL-10, and IL-13 cytokines [5–7].

Th1 and Th2 cytokines are, respectively, known to positive- or negatively regulate macroautophagy [6–15], which is a cell-autonomous effector defense mechanism involved in innate immunity against pathogens (also known as xenophagy), such as *M*. *tuberculosis*, *Leishmania amazonensis*, and human immunodeficiency virus type 1 [8–22]. Macroautophagy (hereafter termed autophagy) is a homeostatic mechanism through which damaged organelles, protein aggregates, and invading intracellular microbes are selectively incorporated into double-membrane vesicles called autophagosomes, followed by vesicle fusion with lysosomes for degradation of its content [23,24]. In the tuberculosis model of infection, the protective role of autophagy and autophagy-related (ATG) proteins have been demonstrated both *in vitro* and *in vivo* [10–18,25]. IFN-γ-mediated autophagy plays a pivotal role in this process, which involves an intermediate member of the Immunity-Related GTPases family (IRG), M protein (IRGM) [10,11,19], and therefore controls *M*. *tuberculosis* infection in MΦs [10–15,26].

The role played by autophagy in the immunopathogenesis of leprosy remains unknown. However, previous studies have shown the presence of *M*. *leprae*-containing double-membrane vacuoles in mouse MΦs, a hallmark of autophagosomes, suggesting a possible involvement of autophagy in the immunomodulatory response of the disease [27,28]. It has also recently been suggested that the *IRGM* gene polymorphism, which is linked with the susceptibility to tuberculosis [29], is associated with an increased risk of developing leprosy by affecting the production of inflammatory cytokines such as IFN-γ [30]. Furthermore, upregulation of *IRGM* was seen in *M*. *leprae*-infected monocytes and MΦs, and was highly expressed in T-lep than L-lep monocytes [31]. A newly published paper has suggested that the killing of *M*. *leprae* in human macrophages was associated with the targeting of mycobacteria to autophagy [32]. Interestingly, several molecules that trigger autophagy, like VDR, TLR2, and NOD2 receptors [13,16,33], are preferentially expressed in T-lep skin lesions [6,34,35], while the polymorphisms in these genes are associated with susceptibility to L-lep [36–38].

Our results demonstrated that autophagy was differentially regulated in T-lep and L-lep patients. T-lep skin lesion cells showed an upregulation of genes related to the *BECN1* (beclin 1, autophagy-related) complex, which, in turn, was associated with increased autophagosome formation and expression of lysosomal peptides. In contrast, we observed that, in L-lep skin lesion cells, *BCL2* (B-cell CLL/lymphoma 2) represented a hindrance to autophagy together with an inhibition of the autophagic flux, restored via IFN-γ treatment. In addition, autophagy was rescued in the cells of L-lep patients who developed type 1 reaction (T1R) episodes. These data suggest that autophagy activation enhances mycobactericidal activity in skin cells, whereas impaired autophagy contributes to an increase in bacillary load.

## Results

### Autophagy is differentially regulated in T-lep *versus* L-lep patients

**Fig 1:**
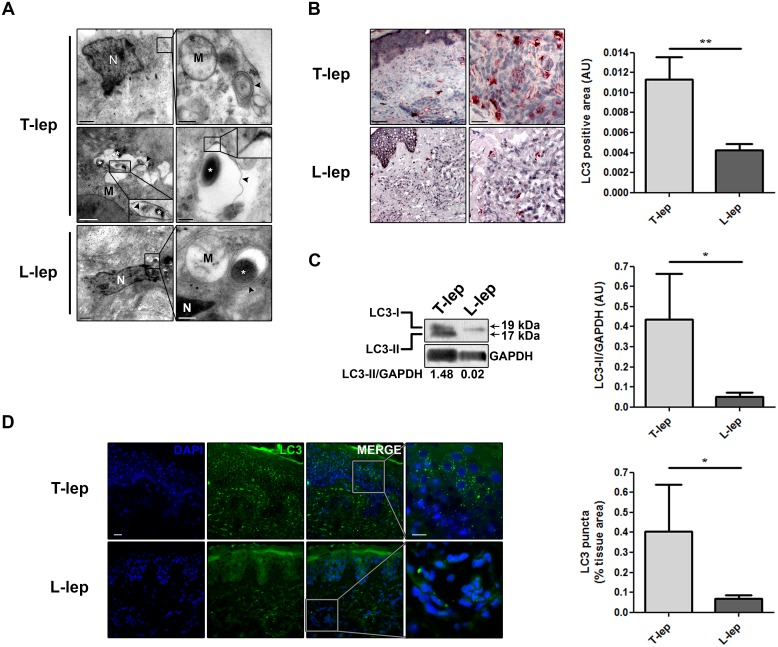
Increase of the autophagy levels in skin lesions of T-lep patients. (A to D) Skin lesion biopsies were obtained from the tuberculoid (T-lep) and lepromatous (L-lep) leprosy clinical forms and analyzed as indicated. (A) Presence of autophagosome-like vacuoles in skin lesion cells of leprosy patients. Representative TEM micrographs from T-lep (n = 3) and L-lep (n = 3) patients are shown. N, nucleus; M, mitochondria; Asterisks, *M*. *leprae*; arrowheads, double-membrane autophagosomes. Scale bars: 0.2 to 1 μm. (B to D) Increased LC3 expression in skin lesion cells of T-lep patients. (B) Immunohistochemical (IHC) analysis of endogenous LC3. Representative micrographs from T-lep (n = 3) and L-lep (n = 4) patients are shown. IHC images were quantified and data are expressed as arbitrary units (AU). Bars represent the mean values ± SEM. **P < 0.01, Mann-Whitney test. Scale bars: 50 and 25 μm. (C) Protein contents from leprosy lesion cells were analyzed by immunoblotting with anti-LC3. GAPDH antibody was used to verify protein amount loading. Representative blots are shown (T-lep, n = 6; L-lep, n = 3). Densitometric analysis of the blots was performed and the LC3-II/GAPDH ratio expressed as AU. Data are presented as mean ± SEM. *P < 0.05, Mann-Whitney test. (D) Redistribution of LC3 by immunofluorescence microscopy. Leprosy tissue sections were immunolabeled with the anti-LC3 (green) and stained with DAPI to visualize the nuclei (blue). Representative micrographs from T-lep (n = 3) and L-lep (n = 3) patients are shown. The number of fluorescent LC3 puncta was quantified and expressed as percentage of tissue area. Data are presented as mean ± SEM. *P < 0.05, Mann-Whitney test. Scale bars: 20 and 10 μm.

In leprosy patients, *M*. *leprae* is found in many cell types and host tissues [2]. To investigate the possible involvement of autophagy in *M*. *leprae* infection, we performed an ultrastructural analysis of T-lep and L-lep skin lesions, to search for double-membrane vacuoles in cells with a morphology similar to that of connective tissue MΦs. Ultrastructural analysis revealed the presence of autophagosome-like vacuoles in skin lesion cells of both T-lep and L-lep patients (Fig 1A). However, the number of empty (Fig 1A, upper micrographs) or *M*. *leprae*-containing (Fig 1A, lower micrographs) autophagosomes was higher in T-lep samples. In addition, we observed double-membrane vacuoles with electron-dense structures resembling damaged bacilli (Fig 1A, lower left micrograph) in T-lep skin lesion cells, but not in L-lep cells.

During autophagy, the nascent proMAP1LC3/LC3 (microtubule-associated protein 1 light chain 3) is processed post-translationally into the cytoplasmatic soluble form LC3-I and by conjugation to phosphatidylethanolamine, generating the membrane-associated LC3-II puncta, which correlates with the extent of autophagosomes [39]. For this reason, LC3 is a commonly-used autophagic marker [40]. In order to confirm if the double-membrane vacuoles observed were autophagosomes, we analyzed the expression of endogenous LC3 by immunohistochemistry to monitor autophagosome formation in leprosy skin lesions. LC3 was strongly expressed in the T-lep lesions in many cell types, including MΦs (Fig 1B). In contrast, LC3 was weakly expressed in only a few cells of L-lep lesions (Fig 1B).

To confirm our data, we performed an immunoblot to measure the proportion of LC3 present in the LC3-II form. We observed a significantly increased amount of LC3-II in T-lep lesions as compared to their L-lep counterparts (Fig 1C). Analysis of the redistribution of endogenous LC3 by immunofluorescence labeling showed that diffuse cytoplasmatic LC3-I was predominant in L-lep lesions in contrast to LC3-II puncta that was prominent in T-lep lesions (Fig 1D). These results indicated that autophagy was involved in the skin immune response against *M*. *leprae* at both leprosy poles; and although it occurred in basal levels in L-lep, it was upregulated in T-lep lesions.

### *M*. *leprae* inhibits autophagosome formation in skin lesion MΦs of L-lep patients

The data gleaned from skin lesion biopsies indicated the activation of autophagy in T-lep patients (Fig 1). However, the cell types that showed enhanced rates of autophagy were not identified in these assays even though many cells showed morphological characteristics compatible with MΦs, the cells specifically targeted by *M*. *leprae* infection. To confirm that MΦs are able to activate autophagy in response to mycobacteria in leprosy lesions, *M*. *leprae*-infected dermal MΦs were obtained directly from fresh skin lesion biopsies, as previously described [41]. The phenotype of the isolated cells from T-lep and L-lep skin lesions was determined by immunofluorescence staining of the human MΦ marker CD68. It was seen that after 7 days of culture more than 95% of the cells were CD68^+^, regardless of clinical form, and that harvested cells expressed both CD68 and LC3 (S1A and S1B Fig).

**Fig 2:**
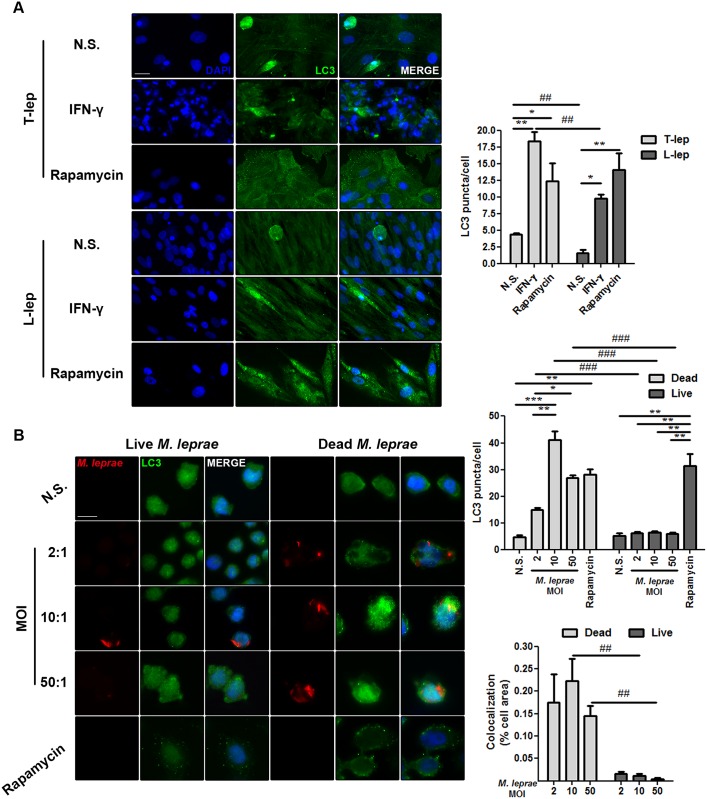
IFN-γ rescues *M*. *leprae*-mediated inhibition of autophagosome formation in skin lesion MΦs of L-lep patients. (A) Macrophages (MΦs) were isolated from skin lesions of tuberculoid (T-lep) and lepromatous (L-lep) patients and treated with IFN-γ (10 ng/mL) or rapamycin (200 ng/mL) for 18 h. Cells were fixed and stained with the anti-LC3 antibody (green) and DAPI (blue). Non-stimulated (N.S.) MΦs from T-lep lesions showed enhanced LC3 puncta formation as compared to L-lep MΦs. IFN-γ treatment increased the number of LC3 puncta in both T-lep and L-lep skin-derived MΦs. The increase was more prominent in T-lep MΦs. A similar increase of LC3-positive vesicles was observed after addition of rapamycin to the cultures of T-lep and L-lep MΦs. Immunofluorescence images were quantified and bars represent the mean values of the number of LC3 puncta per cell ± SEM (T-lep, n = 3; L-lep, n = 3). *P < 0.05, **P < 0.01, Kruskal-Wallis test; ##*P* < 0.01, Mann-Whitney test. Scale bar: 25 μm. (B) Monocyte-derived THP-1 MΦs were infected or stimulated with live or dead PKH26-labeled *M*. *leprae* (red), respectively, at MOIs of 2, 10 and 50 mycobacteria per cell for 18 h. Cells were fixed and immunofluorescence for LC3 (green) and DAPI (blue) was performed. Stimulation with dead *M*. *leprae* directly triggers autophagy. In contrast, infection with live mycobacteria does not induce LC3 puncta accumulation in THP-1 MΦs. Rapamycin (200 ng/mL) treatment was used as a positive control. The number of LC3 puncta/cell was calculated. Colocalization profiles between *M*. *leprae* and LC3 were quantified and expressed as percentage of cell area. Results represent the mean ± SEM of four independent experiments. *P < 0.05, **P < 0.01, ***P < 0.001, Kruskal-Wallis test; ##*P* < 0.01, ###*P* < 0.001, Mann-Whitney test. Scale bar: 10 μm.

Analysis of LC3 puncta formation by immunofluorescence revealed that higher levels of punctate LC3 were detected in skin-derived T-lep MΦs than in L-lep MΦs (Fig 2A). Th1 cytokines are predominantly expressed in T-lep lesions, including IFN-γ, the key cytokine involved in autophagy-mediated control of mycobacteria [5,7,10,19]. To test the hypothesis that the differences in autophagy levels observed among leprosy patients are due to the levels of IFN-γ in skin lesions, the dermal MΦs were treated with recombinant human IFN-γ. As shown in Fig 2A, LC3 puncta accumulated at a remarkable rate in T-lep MΦs in relation to untreated MΦs after IFN-γ treatment. Contrariwise, only a medium number of LC3-positive vesicles were detected in L-lep MΦs when IFN-γ was added to the culture. Yet, the number of vesicles increased over what was found among the non-stimulated MΦs (Fig 2A). Inhibition of the MTOR serine/threonine protein kinase by rapamycin treatment enhanced LC3 puncta formation in both T-lep and L-lep dermal MΦs (Fig 2A), indicating that the decreased levels of autophagy observed in L-lep MΦs were not the result of an intrinsic autophagic defect.

**Fig 3:**
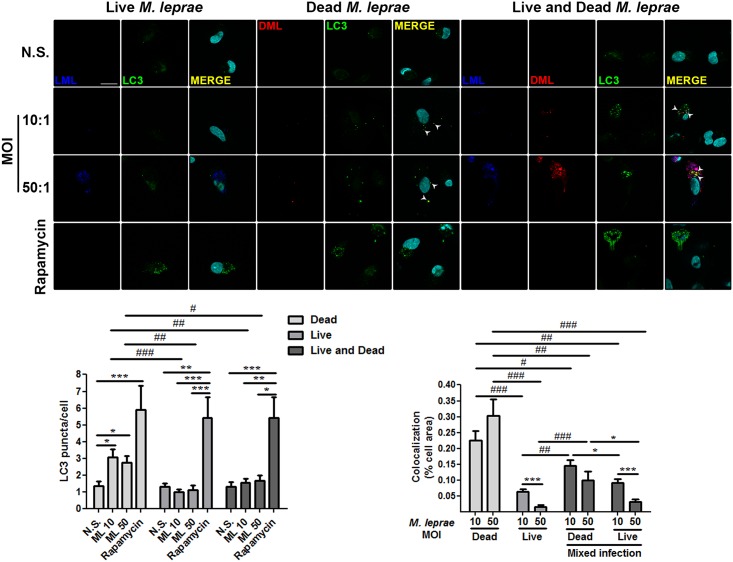
Live *M*. *leprae* inhibits autophagosome formation in primary human monocytes. Blood-derived monocytes from healthy donors were cultured in the presence or absence of live (blue), dead (red), or both live and dead PKH-labeled *M*. *leprae*, at MOIs of 10 and 50 mycobacteria per cell for 18 h. Cells were fixed and immunofluorescence for LC3 (green) and DAPI (cyan) was performed through optical sectioning using structured illumination. LC3 puncta formation in monocytes is inhibited by live, but not dead *M*. *leprae*. Rapamycin (200 ng/mL) treatment was used as a positive control. N.S., Non-stimulated cells. The number of LC3 puncta/cell was calculated. Colocalization profiles between *M*. *leprae* and LC3 were quantified and expressed as percentage of cell area. Results represent the mean ± SEM of three independent experiments. *P < 0.05, **P < 0.01, ***P < 0.001, Kruskal-Wallis test; #*P* < 0.05, ##*P* < 0.01, ###*P* < 0.001, Mann-Whitney test. Scale bar: 20 μm.

Intracellular microorganisms have developed a wide range of strategies to suppress autophagy and enhance their survival, including mycobacteria [24,42–44]. A histological hallmark of L-lep skin lesions is the presence of foamy MΦs containing high amounts of bacilli [2–4]. As shown above, L-lep MΦs displayed less autophagy activation than T-lep MΦs (Fig 2A). We hypothesized that *M*. *leprae* may have a negative effect on autophagy, which is facilitated by a lack of an autophagic stimuli (i.e., IFN-γ) in L-lep lesions. Because the autophagy targeting of *M*. *tuberculosis* and the bacterial countermeasures are dependent processes of active mycobacteria [18,42–45], we infected or stimulated human blood monocytes and THP-1-derived MΦs with live or dead PKH-labeled *M*. *leprae*, respectively. Dead *M*. *leprae* triggered an increase of LC3-positive dots in THP-1 MΦs (Fig 2B) and primary monocytes (Fig 3), but not in a multiplicity of infection (MOI)-dependent manner. At lower bacterial burdens (i.e., MOIs = 2 and 10), we observed dead *M*. *leprae* in close association with LC3-II-decorated autophagosomes in both THP-1 MΦs and blood-derived monocytes (Figs 2B and 3). In higher mycobacterial burdens (i.e., MOI = 50), this colocalization was also detected, but with no significant differences in relation to lower MOIs (Figs 2B and 3). In contrast, higher LC3 puncta levels were not found when THP-1 cells and monocytes were infected with *M*. *leprae*. Furthermore, colocalization between live *M*. *leprae* and LC3-II was less noted in both cell types, and decreases in a MOI-dependent manner (Figs 2B and 3). Indeed, live *M*. *leprae* induced less LC3-positive vesicles than did dead mycobacteria (Figs 2B and 3). We next asked whether live mycobacteria could inhibit the activation of autophagy triggered by dead *M*. *leprae*. For this purpose, we infected blood monocytes with live and dead PKH-labeled mycobacteria simultaneously. We found that during mixed infection, live *M*. *leprae* reduced the number of LC3 puncta similarly to levels observed for infection with live mycobacteria alone (Fig 3). Additionally, in cells infected with both types of bacteria colocalization between *M*. *leprae* and LC3-II was decreased as compared to infection with dead bacteria, and was more prominent with dead than live mycobacteria (Figs 2B and 3). The autophagy inducer rapamycin was used as a positive control, leading to an accumulation of LC3 puncta in both cell types (Figs 2B and 3). Taken together, these findings suggest that reduced levels of autophagy in skin-derived L-lep MΦs are a result of inhibition by *M*. *leprae* infection and that IFN-γ may counteract this effect.

### IFN-γ restores the autophagic flux inhibited by *M*. *leprae* in skin-derived L-lep MΦs

**Fig 4:**
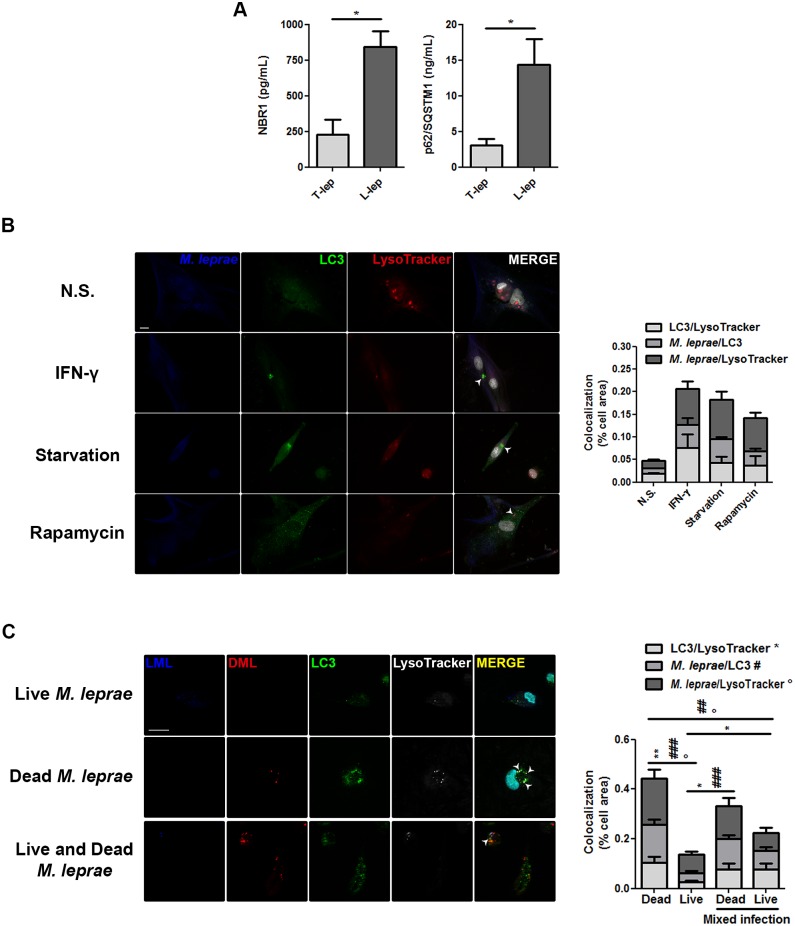
*M*. *leprae*-mediated inhibition of the autophagic flux in skin-derived L-lep MΦs was restored by IFN-γ. (A) Protein contents from skin lesion cells of tuberculoid (T-lep) and lepromatous (L-lep) patients were examined for intracellular levels of SQSTM1/p62 and NBR1 by ELISA. SQSTM1/p62 and NBR1 strongly accumulated in L-lep samples while, in T-lep samples, only low levels were detected. Bars represent the mean values ± SEM of the SQSTM1/p62 (T-lep, n = 5; L-lep, n = 4) and NBR1 (T-lep, n = 6; L-lep, n = 4) levels. *P < 0.05, Mann-Whitney test. (B) Macrophages (MΦs) were isolated from skin lesions of L-lep patients and incubated in full medium with 10 ng/mL IFN-γ or 200 ng/mL rapamycin, or in starvation media (PBS). Eighteen hours after incubation, cells were loaded with 500 nM LysoTracker (red) for 30 min and then fixed and labeled for LC3 (green), *M*. *leprae* LAM (blue), and DAPI (white). In non-stimulated (N.S.) MΦs from L-lep lesions reduced colocalization profiles were seen among LC3, LysoTracker, and LAM, as compared to T-lep MΦs (data in S2 Fig). Activation of autophagy by IFN-γ treatment induces the colocalization of LC3-positive vesicles with LysoTracker stained lysosomes and LAM in both L-lep and T-lep MΦs (data in S2 Fig). Starvation and rapamycin-induced autophagy were also able to promote the autophagic flux in L-lep MΦs. Arrowheads, indicate three-channel colocalization profiles. Colocalization analysis of immunofluorescence images was performed as indicated and expressed as percentage of cell area. Results represent the mean ± SEM of three independent experiments. Scale bar: 10 μm. (C) Blood-derived monocytes from healthy donors were cultured in the presence or absence of live (blue), dead (red), or both live and dead PKH-labeled *M*. *leprae*, at MOI of 50 mycobacteria per cell. Eighteen hours after incubation, cells were loaded with 500 nM LysoTracker (white) for 30 min and then fixed and labeled for LC3 (green) and DAPI (cyan), and imaged through optical sectioning using structured illumination. The autophagic flux in monocytes is inhibited by live, but not dead *M*. *leprae*. Arrowheads, indicate three-channel colocalization profiles. Colocalization analysis of immunofluorescence images was performed as indicated and expressed as percentage of cell area. Results represent the mean ± SEM of three independent experiments. *, #, and °, indicate colocalization profiles between LC3 and LysoTracker, *M*. *leprae* and LC3, and *M*. *leprae* and LysoTracker, respectively. *P < 0.05, **P < 0.01; #*P* < 0.05, ##*P* < 0.01; °*P* < 0.05, Mann-Whitney test. Scale bar: 20 μm.

As previously shown, accumulation of LC3-II was predominant in skin lesion cells of T-lep patients (Figs 1 and 2A). Nonetheless, this accumulation may be due to either an enhanced autophagosome generation or a reduced degradation of the autophagic cargo in the autolysosomes [23,24,40]. To distinguish these steps from each other, we monitored the autophagic flux in skin lesion cells by measuring the intracellular levels of sequestosome 1 (SQSTM1)-like receptors (SLRs). The SLRs SQSTM1 (p62) and NBR1 (NBR1 autophagy cargo receptor) are autophagic adaptors involved in sequestration and degradation of ubiquitinated protein aggregates, misfolded proteins, and bacteria by selective autophagy, at which time SLRs are also degraded, thus serving as a readout of lysosome-dependent autophagic degradation [18,43,46,47]. Dialyzed proteins from leprosy skin biopsies were evaluated by commercial ELISA assays for SQSTM1/p62 and NBR1. Analysis of the intracellular SLRs levels revealed a significant accumulation of both SQSTM1/p62 and NBR1 in L-lep skin lesion cells when compared to the T-lep samples, indicating an impairment in the autophagic flux in L-lep patients (Fig 4A).

The above-mentioned impairment of autophagosomal maturation was further investigated in skin lesion MΦs by fluorescence microscopy. For this purpose, skin-derived MΦs of leprosy patients were submitted to autophagic induction and loaded with acidotropic dye LysoTracker to stain the acidified lysosomes and immunolabeled with specific antibodies for *M*. *leprae* and autophagosomes. As shown in S2 Fig, in untreated T-lep skin MΦs, numerous LC3-positive dots, some of which in juxtaposition to LysoTracker-stained lysosomes and the *M*. *leprae* cell-wall antigen lipoarabinomannan (LAM) were noted. Contrastingly, only a few punctate LC3 could be detected in non-treated L-lep MΦs, and colocalization among LC3, LysoTracker, and LAM was less observed (Fig 4B). Importantly, in both L-lep and T-lep skin lesion MΦs, induction of autophagy with IFN-γ was able to promote the colocalization of LC3-II-decorated autophagosomes with LysoTracker-positive lysosomes and *M*. *leprae* LAM (Fig 4B and S2 Fig). It was also demonstrated that in skin-derived L-lep MΦs, there was a close apposition of LysoTracker with *M*. *leprae*-containing LC3-positive autophagosomes in physiologically- or pharmacologically-induced autophagy (Fig 4B). Besides, via RT-qPCR analysis, T-lep skin lesion cells showed increased mRNA expression of the lysosomal antimicrobial peptides cathelicidin (*CAMP*) and β-defensin 2 (*DEFB4A*) (S3 Fig).

As shown above (Figs 2 and 3), infection with live, but not dead *M*. *leprae* interferes with autophagosome generation, as well as live mycobacteria inhibits the autophagic flux (Fig 4B). Thus, we further investigated whether the mycobacteria viability is required for this blockage of the autophagosomal maturation. We found a decreased formation of autolysosomes in blood monocytes infected with live *M*. *leprae*, as well as reduced targeting of mycobacteria to autophagosomes and lysosomes (Fig 4C). In contrast, dead *M*. *leprae* was highly targeted to autophagosomes and lysosomes in primary monocytes, and autolysosome development was significantly increased in the presence of dead mycobacteria (Fig 4C). Furthermore, in cells infected with both types of bacteria, the autophagic flux was reduced in comparison to infection with dead bacteria, and mycobacterial targeting to autophagy pathway was more notable with dead than live mycobacteria (Fig 4C). As a result, the present results demonstrated that the higher number of accumulated autophagosomes in the skin MΦs of T-lep patients matured into autolysosomes. In L-lep skin MΦs, however, while the autophagic flux is inhibited by *M*. *leprae*, it can be rescued by IFN-γ treatment or other autophagy inducers.

### Gene expression profiles of lesions revealed a differential regulation of autophagy-associated genes in T-lep and L-Lep patients

The results described above indicated that the autophagic response was higher in skin lesion cells, including MΦs, within the group of the self-limited T-lep form versus the disseminated L-lep form (Figs 1, 2 and 4). To further identify the components of the autophagic machinery involved in the cellular response to *M*. *leprae* infection, the transcriptional regulation of *ATG* genes was examined, which may provide correlative data related to autophagy induction [40]. For this purpose, isolated mRNAs of leprosy skin lesions were analyzed by RT-qPCR to obtain gene-expression profiles using a human autophagy pathway PCR array.

**Fig 5:**
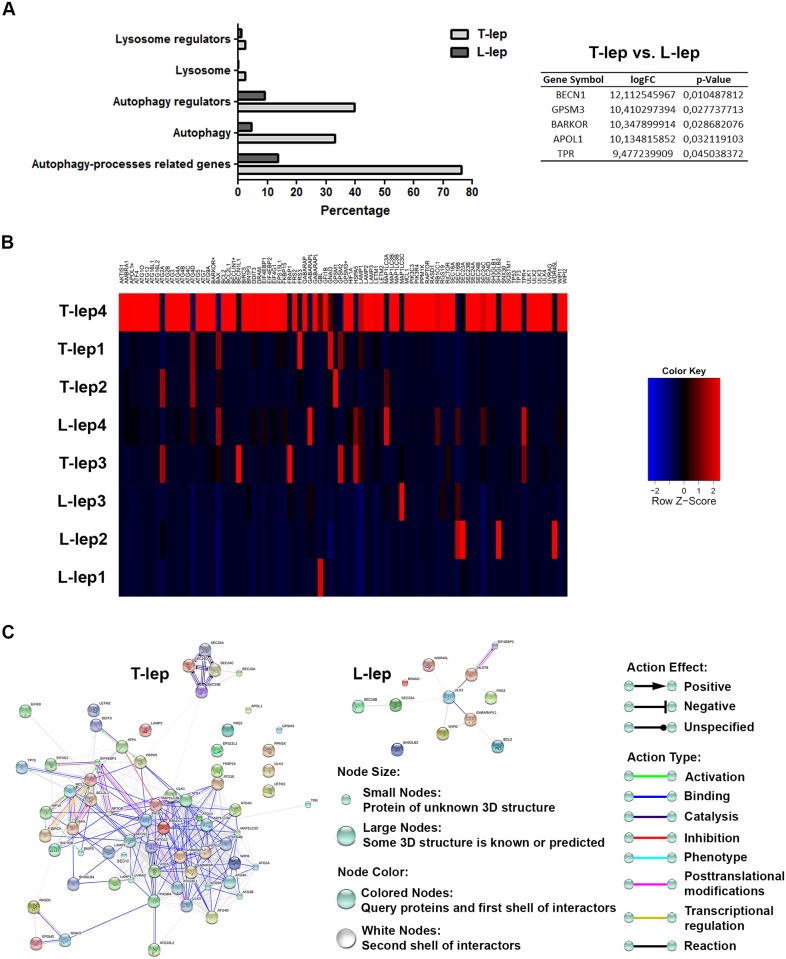
Autophagy gene-expression profiling of leprosy lesions reveals a differential expression of *BECN1* in T-lep patients. (A and B) Purified mRNAs from skin lesions of tuberculoid (T-lep) and lepromatous (L-lep) patients were analyzed by RT-qPCR autophagy array. (A) Autophagy processes-related genes differentially expressed were sub-categorized. The expression fold values of the significantly upregulated genes in T-lep lesions are tabulated (full data are available in S1 Table). Threshold for statistical significance was established at *P* < 0.05. (B) Heat map showing analysis of changes in expression of autophagy processes-related genes in leprosy patients. Each row represents one donor. Asterisks indicate genes with differential expression. Heat map data are representative of four T-lep and seven L-lep samples. (C) Autophagy gene interaction network in T-lep and L-lep skin lesions. Genes with a differential expression in leprosy lesions by autophagy PCR array analysis were visualized by STRING. The action network view. In this view, colored lines and arrow styles between genes indicate the various types of interactions. Network nodes represent genes. Edges represent gene-gene associations. Interactions among autophagy-associated genes were more predominant in T-lep than L-lep patients. Interaction maps are representative of four T-lep and seven L-lep samples.

As shown in Fig 5A and 5B and S1 Table, the gene expression profiles of skin lesion cells revealed a strong upregulation of several autophagy processes-related genes in T-lep (76% of genes) versus L-lep patients (14%) by fold-change analysis. Fold-change upregulated genes in leprosy lesions are involved in autophagosome formation (33% of T-lep genes versus 5% of L-lep genes), regulation of autophagy (40% of T-lep versus 9% of L-lep), lysosomal function or pathways (2% of T-lep versus 0% of L-lep), and regulation of the lysosome (2% of T-lep versus 1% of L-lep) by way of the functional autophagy-lysosomal gene classification previously described by Jegga et al. (Fig 5A and S1 Table) [48].

Moreover, regarding the fold-change analysis, T-lep lesions showed a significantly increased expression in five genes in comparison to those in L-lep lesions (Fig 5B and S1 Table). T-lep lesions were found to have a more highly significant expression of *BECN1*, *GPSM3*, *ATG14* (*BARKOR*), *APOL1*, and *TPR* (Fig 5B and S1 Table). In addition, we also found an increased expression of several other genes located in the core autophagic machinery in T-lep samples such as members of the ATG1/ULK complex (*ULK1/3* and *4*), ATG9 and its cycling system (*ATG2A/B*, *ATG9A* and *WIPI1*), the PIK3 complex (*PIK3C3* and *PIK3R4*), the ATG8 (*ATG3*, *ATG4A/B/C/D*, *ATG7*, *GABARAP*, *GABARAPL2*, *MAP1LC3A/B* and *C*) and ATG12 conjugation systems (*ATG5*, *ATG7*, *ATG10*, *ATG12*, *ATG16L1* and *L2*), and the lysosomal components (*LAMP1/2* and *3*) (Fig 5B and S1 Table).

Conversely, in L-lep lesions fold-change analysis revealed a higher expression of a set of genes mostly involved in autophagy suppression such as *FRS2*, *EIF4EBP2*, *GBL* (*MSLT8*), the anti-apoptotic gene *BCL2*, and others genes such as *ULK2*, the Endophilin B2 gene *SH3GLB2*, *GABARAPL1*, *DRAM* (*DRAM1*), *SEC16B*, *WIPI2*, *WDR45L* (*WDR45B* or *WIPI3*), and *SEC23A* (Fig 5B and S1 Table).

Further, genes differentially modulated in leprosy skin lesions were submitted to gene-gene interactions using the STRING database. Network maps of upregulated genes in leprosy lesions revealed a greater number of interactions between autophagy-associated genes in T-lep than among L-lep patients (Fig 5C and S4 Fig).

**Fig 6:**
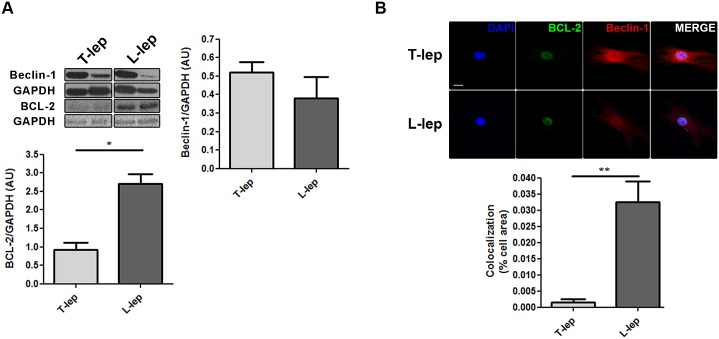
Differential regulation of BECN1 and BCL2 proteins in skin lesion cells of T-lep and L-lep patients. (A) Protein contents from tuberculoid (T-lep) and lepromatous (L-lep) lesion cells were analyzed by western blot with anti-BECN1 and anti-BCL2 antibodies. GAPDH antibody was used to verify protein amount loading. Densitometric band-intensity analysis of the blots was realized and the BECN1/GAPDH (T-lep, n = 6; L-lep, n = 5) and BCL2/GAPDH (T-lep, n = 4; L-lep, n = 4) ratios were expressed as arbitrary units (AU). Bars represent the mean values ± SEM. *P < 0.05, Mann-Whitney test. (B) Macrophages (MΦs) were isolated from skin lesions of T-lep and L-lep patients and cultured for 7 days in full medium. Cells were fixed and labeled for BECN1 (red), BCL2 (green) and DAPI (blue). Cytoplasmatic BCL2 colocalizes with BECN1 in L-lep MΦs, but not in T-lep MΦs. Colocalization profiles between cytosolic BCL2 dots and BECN1 were quantified and expressed as percentage of cell area. The expression of nuclear compartment-associated BCL2 was excluded from the analysis. Results represent the mean ± SEM from one of three immunofluorescence experiments that yielded similar results. **P < 0.01, Mann-Whitney test. Scale bar: 10 μm.

As shown above (Fig 5 and S1 Table), PCR array analysis revealed that pro-autophagic genes were prominently expressed in T-lep lesions. In contrast, the expression of anti-autophagic genes predominated within L-lep lesions. Interestingly, the most upregulated gene in the T-lep lesions was *BECN1*, a key initiator of autophagy in mammals. Whereas in L-lep lesions, a higher expression of *BCL2* was determined, which inhibits autophagy by binding and sequestering BECN1 from the class III PIK3 complex [49]. It was hypothesized that the BECN1/BCL2 relationship might be subject to change during the *M*. *leprae* infection. To investigate this process, the BECN1 and BCL2 protein levels in leprosy skin lesions were assessed by western blot. Unexpectedly, unlike mRNA levels, BECN1 expression was highly variable in leprosy lesions, with no significant differences between T-lep and L-lep patients (Fig 6A). In contrast, BCL2 levels were significantly increased in L-lep versus T-lep lesions (Fig 6A).

The BECN1/BCL2 interaction was further investigated in leprosy skin lesion MΦs by immunofluorescence microscopy. The BCL2 protein has been reported to localize in various cell compartments, including the nuclear envelope, endoplasmic reticulum (ER), and mitochondrial membranes, but only ER-targeted BCL2 is able to inhibit autophagy [49,50]. In skin-derived T-lep MΦs, we observed BCL2 expression mainly at the level of the nuclei in the absence of association with cytoplasmatic BECN1 staining (Fig 6B). However, besides the BCL2 nuclear labeling in skin-derived L-lep MΦs, cytosolic BCL2 dots in colocalization with BECN1 expression were seen, which might represent ER-targeted BCL2 (Fig 6B). Collectively, these results provide evidence that overexpressed BCL2 impaired BECN1-dependent autophagy in L-lep skin lesion MΦs, contributing to the increased bacillary load.

### Type 1 reaction shifts the autophagic profile of L-lep patients

Across the spectrum of leprosy forms, patients may develop complications due to acute episodes of clinical inflammation known as leprosy reactions, classified as type 1 (T1R) or reversal reaction and type 2 reaction or erythema nodosum leprosum [2,51]. L-lep patients with T1R lesions display modifications in their immunological status, shifting away from the former Th2 profile toward a Th1 profile in conjunction with a strong cell-mediated response to *M*. *leprae*, granuloma formation, IFN-γ production, and reduced bacillary load that, overall, resemble T-lep or paucibacillary T1R lesions [2,51].

**Fig 7:**
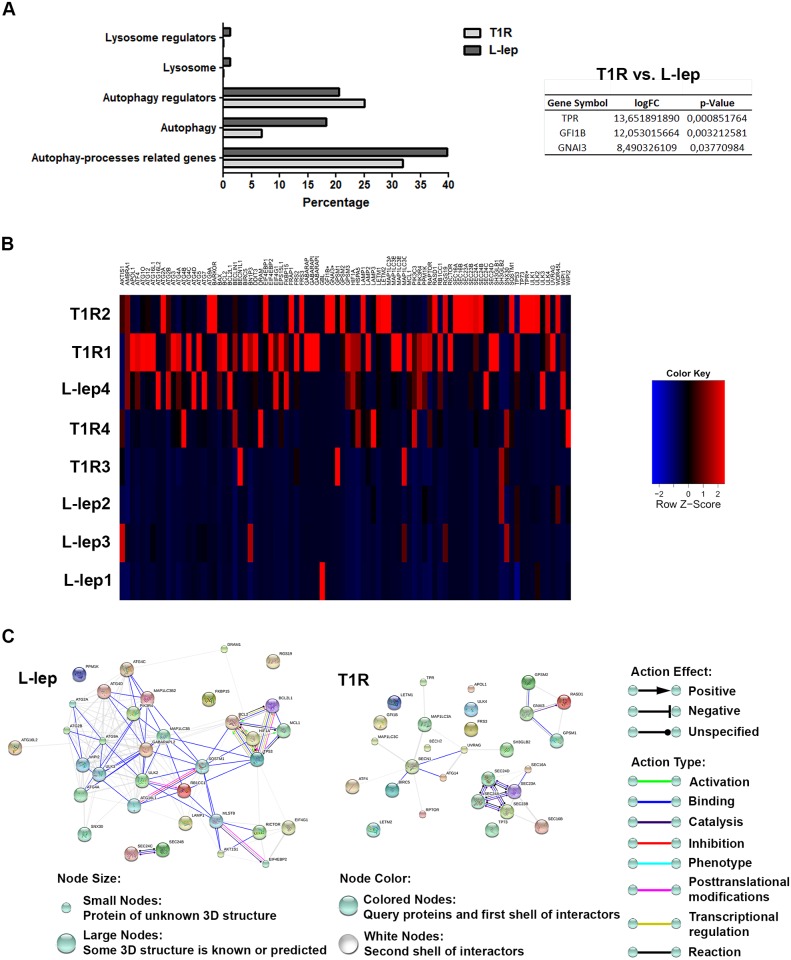
Gene-expression profiles of leprosy lesions showed a balanced modulation of autophagy-associated genes between L-lep patients with and without T1R episodes. (A and B) Purified mRNAs from skin lesions of lepromatous (L-lep) patients with or without type 1 reactional (T1R) episodes were analyzed by RT-qPCR autophagy array. (A) Differentially expressed autophagy processes-related genes were sub-categorized. The expression fold values of the significantly upregulated genes in T1R lesions were tabulated (full data are available in S2 Table). The threshold for statistical significance was *P* < 0.05. (B) Heat map showing analysis of changes in the expression of autophagy processes-related genes in leprosy patients. Each row represents one donor. Asterisks indicate genes with differential expression. Heat map data are representative of seven L-lep and seven T1R samples. (C) Autophagy gene interaction network in L-lep and T1R skin lesions. Genes with a differential expression in leprosy lesions according to autophagy PCR array analysis were visualized by STRING. The action network view. In this view, colored lines and arrow styles between genes indicate the various types of interactions. Network nodes represent genes. Edges represent gene-gene associations. Interactions among autophagy processes-related genes were more evident in L-lep than T1R patients. Interaction maps are representative of seven L-lep and seven T1R samples.

It was then considered whether autophagy activation could be playing a role in this cytokine-driven mycobacterial clearance during multibacillary T1R. To analyze autophagy within this process, the transcriptional regulation of *ATG* genes by RT-qPCR array in L-lep patients with or without T1R episodes was evaluated. As shown in Fig 7A and 7B and S2 Table, the gene expression profiles of skin lesion cells of L-lep and T1R patients revealed a balanced modulation of the genes related to the autophagic processes, showing an increased expression of 40% and 32%, respectively, by fold-change analysis. These upregulated genes are involved in autophagosome formation (18% of L-lep genes versus 7% of T1R genes), regulation of autophagy (20% of L-lep versus 25% of T1R), lysosomal function or pathways (1% of L-lep versus 0% of T1R), and lysosomal regulation (1% of L-lep versus 0% of T1R), as previously described (Fig 7A and S2 Table) [48].

In addition to the fold-change analysis, T1R lesion cells showed a significant upregulation of three autophagy regulators in comparison to L-lep lesions: *TPR*, *GFI1B*, and *GNAI3* (Fig 7B and S2 Table). Interestingly, the five genes significantly upregulated in T-lep lesions (*BECN1*, *GPSM3*, *ATG14*, *APOL1* and *TPR*) are also increased in T1R lesions according to fold-change analysis. Likewise, the three significantly increased genes in T1R patients (*TPR*, *GFI1B* and *GNAI3*) are also upregulated in T-lep patients (Figs 5B and 7B, S1 and S2 Tables).

As shown in Fig 7B and S2 Table, *BCL2L1* was the most upregulated gene in L-lep lesions. As occurs in its familial partner BCL2, also higher in L-lep lesions, BCL2L1 (Bcl-xL) inhibits BECN1-mediated autophagy by blocking the disruption of its binding to BECN1 [49,52]. Furthermore, fold-change analysis revealed an increased expression of others genes such as *ULK2*, *EIF4G1*, and *WIPI2* (Fig 7B and S2 Table). As mentioned above, autophagic genes were similarly modulated in L-lep and T1R patients, revealing an upregulation of key autophagy molecules in both leprosy groups. L-lep lesions were found to have a higher expression of *MAP1LC3B/B2*, *ATG4A/C/D*, *ATG16L1/L2*, *ATG9A*, *GABARAPL2*, *ULK3*, *LAMP1*, *SQSTM1*, *ATG2A*, and *B*. On the other hand, *BECN1L1* (*BECN2*), *MAP1LC3A/C*, and *ULK4* are augmented in T1R lesions (Fig 7B and S2 Table). Although these interactions have also been observed in T1R patients, when analyzed for gene-gene connections through STRING, L-lep lesions prominently showed interactions among modulated autophagy genes. Even so, associations among anti-apoptotic genes were exclusively observed in L-lep samples (Fig 7C and S5 Fig).

As seen above, it was not clear whether the PCR data analysis provided correlative results about autophagy activation in T1R rather than L-lep lesions, as opposed to what was observed regarding T-lep *versus* L-lep lesions (Figs 5 and 7, S1 and S2 Tables). Thus, in order to confirm that the autophagic response increases during the course of T1R episodes in multibacillary leprosy patients, the expression of the autophagy protein LC3 in T1R and L-lep lesions was determined.

**Fig 8:**
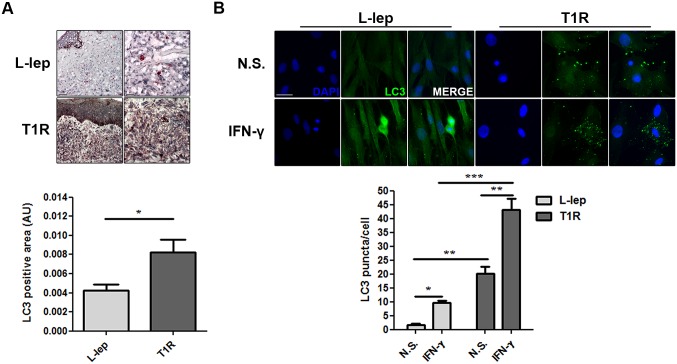
T1R episodes enhances the levels of autophagy in L-lep patients. (A) Immunohistochemistry (IHC) for endogenous LC3. Increase of the LC3 expression in skin lesion cells of L-lep patients undergoing T1R episodes. IHC images were quantified and data are expressed as arbitrary units (AU). Bars represent the mean values ± SEM (L-lep, n = 4; T1R, n = 3). *P < 0.05, Mann-Whitney test. Scale bars: 50 and 25 μm. (B) Macrophages (MΦs) were isolated from skin lesions of L-lep and T1R patients and treated with IFN-γ (10 ng/mL) for 18 h. Cells were fixed and stained with the anti-LC3 antibody (green) and DAPI (blue). Non-stimulated (N.S.) T1R MΦs showed more enhanced LC3 puncta formation than L-lep MΦs. IFN-γ stimulation led to accumulation of LC3 dots in both L-lep and T1R MΦs, but to a lesser extent in MΦs derived from L-lep patients without T1R lesions. Immunofluorescence images were quantified and bars represent the mean values of the number of LC3 puncta per cell ± SEM of three independent experiments. *P < 0.05, **P < 0.01, **P < 0.001, Mann-Whitney test. Scale bar: 50 μm.

Immunohistochemical analysis revealed that the detection of endogenous LC3 was more prominent in L-lep patients undergoing T1R than in non-reactional L-lep lesions (Fig 8A). In addition, immunofluorescence labeling exhibited a higher LC3 puncta formation in skin lesion MΦs of T1R than the L-lep patients (Fig 8B). Upon IFN-γ stimulation, a strong accumulation of large LC3 dots in skin-derived T1R MΦs was apparent. In L-lep skin-derived MΦs, the formation of LC3-positive vesicles was higher after IFN-γ treatment, but to a lesser extent than were T1R MΦs (Fig 8B). Taken together, our results indicated that, similarly to paucibacillary lesions, autophagy is upregulated upon the occurrence of T1R.

## Discussion

The present study details the enrollment of autophagy in the immunopathogenesis of leprosy. We demonstrated that in the multibacillary form of the disease the impairment of the autophagy pathway may significantly contribute to the survival of the bacilli inside the host cells, and it was reversed during T1R episodes.

Recent studies have demonstrated that innate rather than adaptive immunity may be responsible for driving the polarized immune response in leprosy patients. Analysis of the functional pathways related to the MΦ response in leprosy denoted that the MΦ phagocytosis pathway was prominent in the clinically progressive L-lep form, whereas the vitamin D-dependent antimicrobial pathway predominated in the self-limited T-lep form and in patients undergoing T1R [6]. Vitamin D has been implicated in autophagy induction, which, in turn, is associated with the killing of intracellular *M*. *tuberculosis* [10–19,22]. In addition, IFN-γ and TNF, two cytokines reported to increase in the T-lep form of leprosy, are able to induce autophagy. Conversely, IL-10, predominant in the L-lep form, is inhibitory [5–15]. In the present work, we evaluated if autophagy could be differentially regulated in the polar forms of the disease and observed that in the T-lep skin lesion cells, LC3-II expression increased, demonstrating that in these patients autophagy may be responsible for their low bacillary load. In contrast, in L-lep patients, LC3-II expression was reduced. Since the reduced autophagy observed in L-lep patient cells contributed to the increased bacterial load, we tested if the increase could be the result of the cytokine pattern within the microenvironment. Since there are lower levels of IFN-γ in L-lep skin lesions, these cells were stimulated with recombinant human IFN-γ. It was seen that L-lep skin lesion cells were less sensitive to IFN-γ-induced autophagy than were T-lep patient cells although they normally respond to both IFN-γ and rapamycin stimulation.

A recent work has reported that the immunoregulatory cytokine IL-27 was strongly expressed in skin lesions of L-lep patients [53]. Likewise, studies using virulent *M*. *tuberculosis* H37Rv have demonstrated that IL-27 inhibited IFN-γ and starvation-induced autophagy, resulting in the blockage of phagosome maturation and, consequently, the promotion of intracellular mycobacterial survival [54]. In addition, these results linked IL-27 with IL-10 production in *M*. *leprae*-treated macrophages [53]. Since IL-10 is a known autophagic blocker that is upregulated in L-lep lesions, it can be hypothesized that the increased IL-10 production induced by IL-27 in L-lep cells may lead to the reduced sensibility of these cells to IFN-γ-induced autophagy [5,7,9,53,54].

Although reduced in comparison to what occurs in T-lep, autophagy is not fully abrogated in L-lep cells, probably because, at the multibacillary pole of the disease, the inflammatory mediators released by host cells contribute to the equilibrium between roughly 50% dead and 50% viable bacteria in skin lesions. It can be assumed that dead bacteria directly induce autophagy in these cells. We previously demonstrated that IL-10 was responsible for IDO1 induction in cells of lepromatous patients [55]. More recently, the IDO1–EIF2AK4/GCN2 axis has been associated with the induction of autophagy in renal cells, enacting a beneficial role in controlling fatal inflammation in mice [56]. The objective of our future studies is to investigate the IDO1–EIF2AK4/GCN2 axis in L-lep leprosy and the immune regulatory components associated with autophagy induction or blockage.

The present study also demonstrated that dead but not viable *M*. *leprae* induced autophagy in blood monocytes and THP-1 cells. So, it is possible that *M*. *leprae* hampers the autophagy pathway as an immune escape mechanism. Previous works have enunciated that activated macrophages often employ autophagy to eliminate *M*. *tuberculosis*, *Listeria monocytogenes*, *Shigella flexneri*, *Francisella tularensis*, *L*. *amazonensis*, and other pathogens and that the blockage of autophagy may contribute to the growth of these pathogens in host cells [10,20,21,33,42,47]. Although several studies have associated autophagy with *M*. *tuberculosis* killing, previous studies analyzing both pathogenic and non-pathogenic mycobacteria have found that the mycobacterial induction of autophagy varies according to species, taking place independently of MTOR inhibition [57,58].

Since SLRs accumulated when autophagy was inhibited and decreased levels were observed when autophagy was induced, SLRs could be used as markers to study the autophagic flux [40,46]. Its analysis revealed an accumulation of both SQSTM1/p62 and NBR1 in L-lep skin cells. Indeed, in these lesion MΦs, the maturation of autophagosomes without autophagy stimulation was seldom seen. However, despite the observation that *M*. *leprae* may inhibit autophagosome biogenesis, a lower number of mycobacteria might be incorporated into autophagosomes. As such, we are of the opinion that *M*. *leprae* could block both the initiation and maturation steps of autophagy, which is in agreement with our findings shown by fluorescence microscopy, where live *M*. *leprae* inhibits the autophagic flux in blood-derived monocytes. Moreover, we also observed that during mixed infection, live mycobacteria may reduce the autophagic response induced by dead mycobacteria, and that dead *M*. *leprae* is preferentially targeted to the autolysosomes. This finding is consistent with a recent report, which showed a selective resilience to the maturation step of autophagy in autophagosomes that contains virulent, but not avirulent *M*. *tuberculosis* [59]. Nonetheless, in precisely what ways *M*. *leprae* inhibits autophagy and how MΦs are empowered to target *M*. *leprae* as an autophagic cargo destination remain unclear. We hope to elucidate both questions in the near future.

An array analysis of autophagy was a contributing factor in deciphering the pathways that could be differentially modulated in T-lep and L-lep skin cells. In T-lep lesions, increased gene expression associated with autophagosomal formation and maturation as well as autophagy and apoptosis regulation was observed. In T-lep lesions, there was a predominance of proinflammatory cytokines that may contribute to both apoptosis and autophagic induction. The regulation of these two pathways determines the fate of the infection. Our data suggested that the autophagy induced by these proinflammatory cytokines in T-lep lesions may contribute to mycobacterial killing. In contrast, apoptosis contributes to the persistence of infection. In this connection, our previous work demonstrated that even though the MΦ1 phenotype was predominant in T-lep lesions, efferocytosis contributed to the maintenance of a minimum of MΦ2 cells, which are essential for sustaining the infection in leprosy skin lesions [4].

The gene with the highest fold-change expression in the analysis of T-lep and L-lep lesions was *BECN1*, essential in the functional formation of autophagosomes [49,60]. Interestingly, BECN1 expression may be induced by IFN-γ to activate autophagy [61]. *ATG14* expression was also significantly higher in T-lep lesions. Other authors have demonstrated that ATG14 was important in directing BECN1 toward autophagosomes and, along with BECN1, were also a prerequisite for autolysosome formation [62,63]. *APOL1*, another significantly overexpressed gene also associated with autophagic induction, is induced by IFN-γ and TNF, two upregulated cytokines in T-lep lesions [5,64]. Interestingly, it was recently demonstrated that like the *S100A12* antimicrobial gene, *APOL1* is a constituent of the IFN-γ inducible human macrophage host defense network against *M*. *leprae* [65].

In L-lep lesions, *BCL2* was upregulated, as previously described [66]. Studies with the intracellular pathogen *Coxiella burnetti* have demonstrated that this pathogen may modulate autophagy as well as apoptosis pathways through BECN1/BCL2 interplay modification to generate a persistent bacterial infection in the host cells [67]. It has also been shown that cells stably expressing BCL2 could inhibit starvation-induced autophagy via disruption of the BECN1/class III PIK3 complex or antagonisms of other as-of-yet-defined BECN1 activities that are necessary for autophagy [49,67]. It has recently been proposed that the autophagy inhibition mediated by anti-apoptotic BCL2 family members requires dissociation of the BECN1 binding, and occurs independently of the pro-apoptotic BCL2 family members BAX and BAK1 [68]. Thus, our data suggest that BECN1 mediates autophagy in T-lep cells but that BCL2 may inhibit this effect in L-lep cells.

In T-lep lesions, increased expression of mitochondrial stress genes (i.e., *SH3GLB1*, *ATG4D* and *BNIP3*) was noted while an ultrastructural analysis did not reveal excessively damaged mitochondria or signals of mitophagy. Parkin-dependent mitophagy is antagonized by pro-survival members of the BCL2 family, BCL2L1, and MCL1 [69]. Both *BCL2L1* and *MCL1* were upregulated in T-lep cells, which may explain the absence of mitophagy in T-lep lesions.

Patients with the borderline or anergic subpolar lepromatous forms may develop T1R during or ending multidrug therapy. T1R is a challenging problem because it is the most important cause of nerve damage in leprosy leading to the feared disabilities and deformities associated with the disease [2,51]. It is hypothesized that in the T1R forms, skin immune cells such as MΦs become spontaneously activated, thus initiating a local innate immune response against the existing *M*. *leprae* components, overwhelming the predominant immunosuppressive state. Several studies have demonstrated that IFN-γ-induced genes play an important role in T1R [65,70,71]. Since IFN-γ was able to restore autophagy activation in the skin cells of lepromatous patients, we hypothesized that higher IFN-γ levels may contribute to the autophagic induction leading to bacterial killing by macrophages and the concomitant inflammatory status observed in T1R skin lesions. Three autophagy regulators were significantly upregulated in T1R in comparison to what was found in the L-lep lesion cells. Moreover, all significantly upregulated genes in T-lep increased in T1R as well. These genes increased in both T-lep and T1R were associated with different pathways, including BECN1- and TNF-mediated autophagy.

As a whole, our results demonstrated that *M*. *leprae* impaired host cell autophagy as an immune escape mechanism, which, however, was counteracted by IFN-γ stimulation. Proinflammatory cytokines may induce autophagy in T-lep lesions by a process dependent on BECN1 and APOL1. In L-lep cells, BCL2 family members impaired BECN1-mediated autophagy induction; and, in L-lep cells, T1R was characterized by an autophagic rescue. In conclusion, the overall results of the present study suggested that proteins from autophagy pathway could be viewed as targets for drug development and immunoprophylaxis in leprosy.

## Materials and Methods

### Patients and clinical specimens

The leprosy patients involved in the study were recruited from the Souza Araújo Outpatient Unit (FIOCRUZ) and classified according to Ridley and Jopling criteria [3]. All classified T-lep patients were diagnosed as borderline tuberculoid and all L-lep patients had lepromatous polar leprosy (LL). The skin biopsies of the T-lep and L-lep patients were taken at diagnosis and, thus, prior to treatment. Reversal reaction biopsies were taken during T1R episodes in patients originally diagnosed with borderline lepromatous (BL). Because the T1R group claimed BL background in the PCR array analysis, BL patients were allocated within the L-lep group for comparison. The clinical and demographic data of all patients recruited in this study are presented in the Table 1 and S3 Table.

**Table 1:** Baseline characteristics of the patients with leprosy included in the study.

	T-lep	L-lep	T1R
**Characteristic**			
Male/Female, n	14/12	22/6	7/4
Age, Mean (range)	51 (20–69)	45,71 (21–73)	53 (26–70)
BI, Mean (range)	0 (0–0)	4,33 (0,50–5,85)	1,45 (0–3,75)
LBI, Mean (range)	0 (0–0)	5,23 (2,70–5,90)	2,35 (0–3,80)
**Ridley—Jopling Clinical Form of Leprosy, n**			
BT	26	-	-
BL	-	3	11
LL	-	25	-
**Leprosy Treatment Status, n**			
Pretreatment	26	28	2
On treatment	-	-	9

### Ethics statement

All recruited leprosy patients were approved by the Institutional Ethics Committee of FIOCRUZ (number 1.538.467). Written informed consent was obtained from all patients before inclusion in the study. Buffy coats from healthy donors were obtained at the hemotherapy service of the Clementino Fraga Filho University Hospital (Federal University of Rio de Janeiro).

### Skin lesion macrophages isolation

Skin lesion biopsies from the T-Lep, L-Lep and T1R patients were collected onto 15 mL conical centrifuge tubes (Corning) containing RPMI 1640 (GIBCO) at 4°C; and the cells were isolated, as previously described by Moura et al. 2007 [41]. Briefly, the dermis was fragmented into little pieces and placed in perfluoroalkoxy vials (Chemware) containing RPMI 1640, 10% Fetal Bovine Serum (FBS; GIBCO), 4 mg/mL dispase II (GIBCO), and 0.5 mg/mL collagenase type I (GIBCO) and incubated overnight at 37°C in a humid atmosphere of 5% CO_2_. After digestion, the cell suspensions were passed through a 70 μm nylon mesh cell strainer (BD Falcon) and then washed three times with RPMI 1640 by centrifugation at 500 *g* for 10 min at 4°C. Pelleted cells were resuspended in full nutrient medium: RPMI 1640 supplemented with 10% FBS, 2 mM L-alanyl-L-glutamine (GlutaMAX I, GIBCO), and 100 μg/mL ampicillin (Sigma-Aldrish). Cells were plated at 1 x 10^5^ cells/mL on 15 mm sterile circular coverslips (Glasscyto) and cultured for 7 days at 37°C.

After this period, nonadherent cells were removed and MΦs were immunophenotyped by fluorescence microscopy and checked for mycobacterial presence by Ziehl-Neelsen acid-fast bacilli stain. The number of CD68^+^ cells was quantified using the Cell Counter plugin of ImageJ 1.50g image analysis software (Wayne Rasband, National Institutes of Health), with each experiment involving the scoring of at least 100 cells per sample.

### Cell culture

The human monocytic cell line THP-1 was obtained from the American Type Culture Collection (ATCC) and differentiated to MΦs with 200 nM Phorbol 12-Myristate 13-Acetate (Sigma-Aldrich) for 24 h at 37°C. Peripheral blood mononuclear cells were isolated from buffy coats using Ficoll-Paque PLUS (GE Healthcare) density gradient. Blood-derived monocytes were purified by plastic adherence for two hours in serum-free RPMI 1640. Non-adherent cells were removed via strong washing with warm Phosphate-Buffered Saline (PBS; Sigma-Aldrich). Cells were cultivated at 5 x 10^5^ cells/mL on 15 mm sterile circular coverslips in RPMI 1640 supplemented with 10% FBS, 2 mM L-alanyl-L-glutamine, and 100 μg/mL ampicillin (for live mycobacteria infection) or 100 U/mL penicillin and 100 μg/mL streptomycin (GIBCO).

### *M*. *leprae* infection

*M*. *leprae* Thai-53 isolate was maintained by serial passage in the footpads of athymic nude mice; and mycobacteria was harvested aseptically and analyzed for viability using a method described elsewhere [72]. Dead *M*. *leprae* was generated by exposition to 10^6^ rad of gamma irradiation from a ^60^Co source. In order to obtain fluorescent *M*. *leprae*, the bacterial suspensions were labeled via the PKH26 Red or the PKH67 Green Fluorescent Cell Linker Kits (Sigma-Aldrich) according to the manufacturer’s instructions. Cells were infected with live or stimulated with γ-irradiated (dead) PKH-labeled *M*. *leprae* at MOIs of 2:1, 10:1, or 50:1 organisms/cell and exposed for 18 h at either 33°C or 37°C in 5% CO_2_, respectively. For mixed infection, monocytes were incubated at 33°C in 5% CO_2_.

### Induction of autophagy

Autophagy was triggered by treatment with recombinant IFN-γ (10 ng/mL; BD Pharmingen) or rapamycin (200 ng/mL; Assay Designs) for 18 h at 37°C in full nutrient medium [13,20]. Alternatively, autophagy was induced by amino acid and serum starvation through incubation of cells in PBS at 37°C for 18 h [10,20].

### Transmission electron microscopy

Skin lesion biopsies from T-Lep and L-Lep patients were fixed with 2.5% glutaraldehyde in 0.2 M cacodylate buffer pH 7.2 for 24 h at 4°C, and postfixed in 2% osmium tetroxide in 0.1 M cacodylate buffer for 1 h at 4°C. The skin biopsies were then dehydrated in graded acetone, embedded in Epoxy resin (EMbed-812 kit), and polymerized at 60°C for 72 h. Ultrathin 70–80 nm sections were collected on 300-mesh copper grids, stained with uranyl acetate and 2% lead citrate (Lead Nitrate; Sodium Citrate, Grupo Química), and viewed using a JEOL JEM-1011 transmission electron microscope (JEOL, Tokyo, Japan) operating at 60 kV beam voltage. All reagents were purchased from Electron Microscopy Sciences unless otherwise specified. Images were digitally captured with a SC1000 ORIUS CCD camera (Gatan Inc., California, USA) using Gatan Digital Micrograph 2.31.734.0 software (Gatan Inc.).

### Immunohistochemistry

Skin lesion frozen section biopsies of T-Lep, L-Lep, and T1R patients were performed in a Leica LM3000 cryostat (Leica, Wetzlar, Germany). The cryostat sections (4 μm thick) were fixed in acetone, hydrated in 0.01 M Ca^2+^Mg^2+^-free PBS, and incubated with 0.3% hydrogen peroxide (Vetec) in PBS for 10 min to quench endogenous peroxidase activity. Unspecific binding sites were blocked with normal horse serum (VECTASTAIN Elite ABC Kit Mouse IgG, Vector Laboratories) for 30 min at room temperature. The mouse anti-human LC3 mAb antibody (MBL International, M152-3) was diluted 1:50 in PBS 0.25% Triton X-100 (Sigma-Aldrich) and incubated for 1 h at room temperature. The sections were washed three times with PBS containing 0.25% Triton X-100 and incubated with biotinylated horse anti-mouse IgG (VECTASTAIN Elite ABC Kit) for 1 h at room temperature. After washing, the sections were incubated for 40 min with avidin DH–biotinylated horseradish peroxidase (HRP) H complex (VECTASTAIN Elite ABC Kit) for signal amplification. The reaction was developed at room temperature in a solution of 3-amino-9-ethylcarbazole (AEC Peroxidase HRP Substrate Kit, Vector Laboratories) for 10 min.

Slides were counterstained with Mayer’s haematoxylin (Dako), mounted with aqueous Faramount mounting medium (Dako), and viewed through a Nikon Eclipse E400 microscope with a plan-apochromat 40x/0.65 objective (Nikon Instruments Inc., New York, USA). Images were acquired by using the INFINITYX-32C camera and Infinity Capture software 6.1.0 (Lumenera Corporation, Ontario, Canada). LC3-positive area expressed in the graphs as arbitrary units (AU) was assessed by the ratio between labeled and total tissue area sum values obtained after the selection of the interest areas using the tool “Count/Size” and the function “Statistics” in the software “Image-Pro Plus 6.0” (Media Cybernetics, Inc., Rockville, USA). A minimum of ten random images per sample were evaluated for each experiment.

### Western blot

Proteins were extracted from the organic phase of TRIzol-treated (Life Technologies) T-Lep and L-Lep skin lesion biopsies by protein dialysis, as recommended by the manufacturer. Equivalent amounts of protein (20 μg) from T-Lep and L-Lep biopsies were resolved on 12% polyacrylamide gels by SDS-PAGE. Resolved proteins were transferred to Hybond-C Extra nitrocellulose membranes (Amersham Biosciences) in 25 mM Tris (Bio-Rad), 190 mM glycine (Bio-Rad) and 20% methanol (Merck) using an electrophoretic transfer system with cold-block (Bio-Rad, California, USA). The membranes were blocked using 5% bovine serum albumin (BSA; Sigma-Aldrich) in TBS containing 0.1% Tween-20 (Calbiochem) at room temperature for 1 h. After blocking, the membranes were immunoblotted sequentially overnight with primary antibodies against LC3 (1:400; MBL International), BECN1 (1:1000; rabbit IgG anti-human, Assay Designs, 905-721-100), BCL2 (1:200; mouse IgG1 anti-human, Dako, M088729) and Glyceraldehyde-3-Phosphate Dehydrogenase (GAPDH; 1:500; mouse IgG1 anti-human, Santa Cruz Biotechnology, sc-47724). Incubations were followed by washing; and bound antibodies were detected with the appropriate HRP-conjugated secondary antibodies goat anti-mouse IgG-HRP (1:2000; DakoCytomation, P0447) or goat anti-rabbit IgG-HRP (1:2000; DakoCytomation, P0448) for 1 h at room temperature.

Immuno-reactive bands were detected using the chemoluminescent substrate western blotting Luminol reagent (Santa Cruz Biotechnology), and revealed using medical X-ray film (Carestream Kodak X-Omat LS film, Amersham Biosciences) or the Carestream Image Station 4000MM (Carestream Health, Connecticut, USA). Densitometric analysis was performed using Adobe Photoshop CS6 software (Adobe Systems Incorporated, USA).

### Immunofluorescence assay

T-Lep and L-Lep skin lesion frozen section biopsies were performed in a Leica LM3000 cryostat, fixed in acetone and hydrated in 0.01 M Ca^2+^Mg^2+^-free PBS. Unspecific binding sites were blocked with 10% Normal Goat Serum (NGS; Sigma-Aldrich) and 0.01% nonfat dried milk in PBS and 2 drops of Protein Block Serum-Free Ready-To-Use (Dako) at room temperature for 30 min. The LC3 mAb antibody (1:50; MBL International) was diluted in 2% NGS, PBS containing 0.25% Triton X-100 and incubated for 1:30 h at room temperature. Tissue sections were washed three times and incubated with Alexa Fluor 488 goat anti-mouse IgG secondary antibody (1:1000; Molecular Probes, A11001) for 1:30 h at room temperature. The nuclei were stained with 4’-6-diamidino-2-phenylindole (DAPI; 1:10000, Molecular Probes) and slides were mounted with VECTASHIELD mounting medium (Vector Laboratories).

For cell culture, monolayers were fixed with 4% paraformaldehyde (Sigma-Aldrich) for 20 min at 4°C. Cells were then washed three times with PBS 0.01% Triton X-100 (Sigma-Aldrich) and blocked with 10% FBS, 10% NGS, and 1% BSA for 1 h at room temperature. The buffer was then removed and the following primary antibodies were added: mouse IgG1 anti-human LC3 (1:50; MBL International), mouse IgG2b anti-human CD68/SR-D1 (1:100; R&D Systems, MAB20401), rabbit IgG anti-LAM (1:50; kindly donated by Dr. John Stewart Spencer from the Mycobacteria Research Laboratories, Colorado State University), rabbit IgG anti-human BECN1 (1:500; Assay Designs), and mouse IgG1 anti-human BCL2 (1:100; Dako), and incubated overnight at 4°C. Afterwards, the cells were washed and incubated with the secondary antibodies Alexa Fluor 532 goat anti-rabbit IgG (1:500, Molecular Probes, A11009), Alexa Fluor 546 goat anti-mouse IgG2b (1:500; Molecular Probes, A21143), or Alexa Fluor 633 goat anti-mouse IgG1 (1:500; Molecular Probes, A21126) for 2 h at room temperature. For lysosome staining the acidotropic dyes LysoTracker Blue DND-22 or Yellow HCK-123 (500 nM; Molecular Probes) were added to the cultures 30 min before fixation. Finally, nuclei were stained with DAPI. The coverslips were mounted with PermaFluor Aqueous Medium (Thermo Scientific).

Tissues and cells were imaged using an Axio Observer. Z1 microscope equipped with the Colibri.2 and ApoTome.2 illumination systems (Carl Zeiss, Oberkochen, Germany) and the EC Plan-Neofluar 40x/1.30, 63x/1.40, and 100x/1.30 oil objectives. Images were acquired with a digital camera AxioCam HRm and AxioVision Rel. 4.8.2.0 software (Carl Zeiss). The number of fluorescent LC3 puncta was quantified using the Particle Analyzer plugin of ImageJ software after image thresholding [14,32]. ImageJ was also used for colocalization analysis. Briefly, the red, green, and blue channels were extracted from RGB images and converted to binary images (grayscale) by automatic thresholding. Afterwards, the background pixelation was removed from the analysis, and then merged channels were analyzed using the Colocalization and Analyze Particles built-in functions of the software [32]. For both analysis, a minimum of 100 cells per sample were scored by each experiment.

### Intracellular ELISA

To access the SLRs protein levels in skin lesion cells, dialyzed proteins (10 μg) from T-Lep and L-Lep biopsies were measured by commercial ELISA kits for SQSTM1/p62 and NBR1 (Enzo Life Sciences), as described by the manufacturer.

### RNA isolation, reverse transcription, and real time PCR quantifications

RNA was extracted from T-Lep, L-Lep and T1R skin lesion biopsies by the TRIzol method according to the manufacturer’s instructions. RNA samples were treated with DNAse to avoid gDNA contamination (RTS DNase Kit, MO BIO Laboratories); and RNA integrity was analyzed by 1.2% agarose gel electrophoresis (UltraPure agarose, Life Technologies). One microgram of RNA from leprosy lesions was reverse transcribed using the SuperScript III First Strand Synthesis System (Life Technologies). Real time gene expression was performed by way of a human autophagy PCR array (Real Time Primers, HATPL-I) composed of 88 autophagy-associated targets and 8 reference genes (*ACTB*, *B2M*, *GAPDH*, *GUSB*, *HPRT1*, *PGK1*, *PPIA*, and *RPL13A*). The full list of genes is available on http://realtimeprimers.com/huauprli.html, and in S1 and S2 Tables. The qPCR autophagy array was conducted under the PCR conditions recommended by the manufacturer and Power SYBR Green PCR Master Mix (Applied Biosystems). Alternatively, the TaqMan Fast Universal PCR Master Mix (Applied Biosystems) was used to determine mRNA expression of *DEFB4A* (ThermoFisher Scientific, Hs00823638_m1) and *CAMP* (ThermoFisher Scientific, Hs00189038_m1).

qPCR reactions were performed in a StepOnePlus Real-Time PCR System (Applied Biosystems, MA, USA). Gene expression data were analyzed by the 2^−ΔCT^ method and normalized using the housekeeping gene Hypoxanthine Phosphoribosyltransferase 1 (*HPRT1*; Real Time Primers) for autophagy arrays, or the reference gene *GAPDH* (Hs02758991_g1, ThermoFisher Scientific) for TaqMan assays.

### Autophagy pathway analysis

Gene expression profiles of leprosy lesions were derived from the RT-qPCR autophagy array. The Linear Model for Series of Arrays (lmFit) and Empirical Bayes Statistics for Differential Expression (ebayes) functions from the “limma” (Bioconductor) R package were used to measure differences in gene expression. Genes that were expressed differentially in the two leprosy groups were identified by log2 fold change (≥ 1.5-fold) and moderated t-test *P* value (*P* < 0.05) thresholds [6,35]. Autophagy processes-related genes differentially expressed were functionally sub-categorized into four subgroups (autophagy, autophagy regulators, lysosome, and lysosome regulators) according to a set list of human gene symbols involved in autophagy and lysosomal pathways [48]. Heat maps were generated using the Enhanced Heat Map (heatmap.2) function from the “gplots” R package and displayed in a z-scores scaling.

### Gene interaction analysis

In autophagy pathway analysis, the differentially modulated genes were evaluated by the Search Tool for the Retrieval of Interacting Genes/Proteins (STRING) 10.0 database (http://string-db.org/). Network maps of gene-gene interactions in leprosy lesions were obtained through action and confidence views of STRING.

### Statistical analysis

Statistical significance was calculated by Mann-Whitney or Kruskal-Wallis tests using GraphPad Prism 5.04 software (GraphPad, La Jolla, CA, USA). A *P* value < 0.05 was considered statistically significant.

## Supporting Information

S1 Fig CD68 expression in skin lesion MΦs of leprosy patients. (A and B) Macrophages (MΦs) were isolated from skin lesions of tuberculoid (T-lep) and lepromatous (L-lep) patients and cultivated for 7 days in full medium. Cells were fixed and stained as indicated. DAPI was used to visualize the nuclei (blue). (A) Skin-derived MΦs of T-lep and L-lep patients did not show any significant differences in CD68 expression (red). The percentages of CD68^+^ cells in immunofluorescence are shown. Data are presented as mean ± SEM (T-lep, n = 3; L-lep, n = 3). Scale bar: 25 μm. (B) Coexpression of CD68 (red) and LC3 (green) in skin lesion MΦs of leprosy patients. The images are representative of a T-lep patient. Scale bar: 10 μm.
(TIF)

S2 Fig Normal autophagosomal maturation process in skin-derived T-lep MΦs. Macrophages (MΦs) were isolated from skin lesions of T-lep patients and incubated in full medium with 10 ng/mL IFN-γ. Eighteen hours after incubation, cells were loaded with 500 nM LysoTracker (red) for 30 min and then fixed and labeled for LC3 (green), *M*. *leprae* LAM (blue) and DAPI (white). Fusion profiles between LysoTracker-labeled lysosomes and *M*. *leprae-*containing LC3-positive autophagosomes were observed in both non-stimulated (N.S.) and IFN-γ-treated MΦs of T-lep patients. Arrowheads, indicate three-channel colocalization profiles. The images are linked to the experiments described in Fig 4B. Colocalization analysis of immunofluorescence images was performed as indicated and expressed as percentage of cell area. Results represent the mean ± SEM of three independent experiments. Scale bar: 20 μm.
(TIF)

S3 Fig Increase of mRNA encoding the antimicrobial peptides cathelicidin and β-defensin 2 in T-lep lesions. Purified mRNAs from skin lesions of tuberculoid (T-lep) and lepromatous (L-lep) patients were analyzed for cathelicidin (CAMP) and β-defensin 2 (DEFB4A) expression by RT-qPCR. Gene expression data were normalized to GAPDH and mRNA levels are expressed as arbitrary units (AU). Bars represent the mean values ± SEM of six independent samples of each patient group. *P < 0.05, **P < 0.01, Mann-Whitney test.
(TIF)

S4 Fig Autophagy gene interaction network in T-lep and L-lep skin lesions. Genes with a differential expression in leprosy lesions by autophagy PCR array analysis were visualized by STRING. The confidence network view. In this view, the color thickness of the edges represents the confidence score of a functional association. Network nodes represent genes. Edges represent gene-gene associations. Interactions among autophagy-associated genes were more predominant in tuberculoid (T-lep) than lepromatous (L-lep) patients. Gene networks are linked to the experiments described in Fig 5. Interaction maps are representative of four T-lep and seven L-lep samples.
(TIF)

S5 Fig Autophagy gene interaction network in L-lep and T1R skin lesions. Genes with a differential expression in leprosy lesions according to autophagy PCR array analysis were visualized by STRING. The confidence network view. In this view, the color thickness of the edges represents the confidence score of a functional association. Network nodes represent genes. Edges represent gene-gene associations. Interactions among autophagy processes-related genes were more evident in lepromatous (L-lep) than type 1 reaction (T1R) patients. Gene networks are linked to the experiments described in Fig 7. Interaction maps are representative of seven L-lep and seven T1R samples.
(TIF)

S1 Table Autophagy pathway gene expression analysis in T-lep and L-lep skin lesions. Purified mRNAs from skin lesions of tuberculoid (T-lep) and lepromatous (L-lep) patients were analyzed by RT-qPCR autophagy array. Differentially expressed autophagy processes-related genes between the leprosy groups were identified by fold change (≥ 1.5-fold) and moderated t-statistic (*P* < 0.05) using the empirical Bayes approach in R software and then sub-categorized. Full names, categories, expression fold, and *P* values of the upregulated genes in T-lep and L-lep lesions were tabulated. Table data are linked to the experiments described in Fig 5. PCR array data are representative of four T-lep and seven L-lep samples.
(XLSX)

S2 Table Autophagy pathway gene expression analysis in L-lep and T1R skin lesions. Purified mRNAs from skin lesions of lepromatous (L-lep) and type 1 reaction (T1R) patients were analyzed by RT-qPCR autophagy array. Differentially expressed autophagy processes-related genes between the leprosy groups were identified by fold change (≥ 1.5-fold) and moderated t-statistic (*P* < 0.05) using the empirical Bayes approach in R software and then sub-categorized. Full names, categories, expression fold, and *P* values of the upregulated genes in L-lep and T1R lesions are tabulated. Table data are linked to the experiments described in Fig 7. PCR array data are representative of seven L-lep and seven T1R samples.
(XLSX)

S3 Table Baseline characteristics of the patients with leprosy included in each experiment of the study. (XLSX)
