# Supplementary figures and images for: Autophagy Is an Innate Mechanism Associated with Leprosy Polarization
Source: PLoS Pathog. 2017 Jan 5;13(1):e1006103. doi: 10.1371/journal.ppat.1006103 (PMC5215777; doi:10.1371/journal.ppat.1006103)

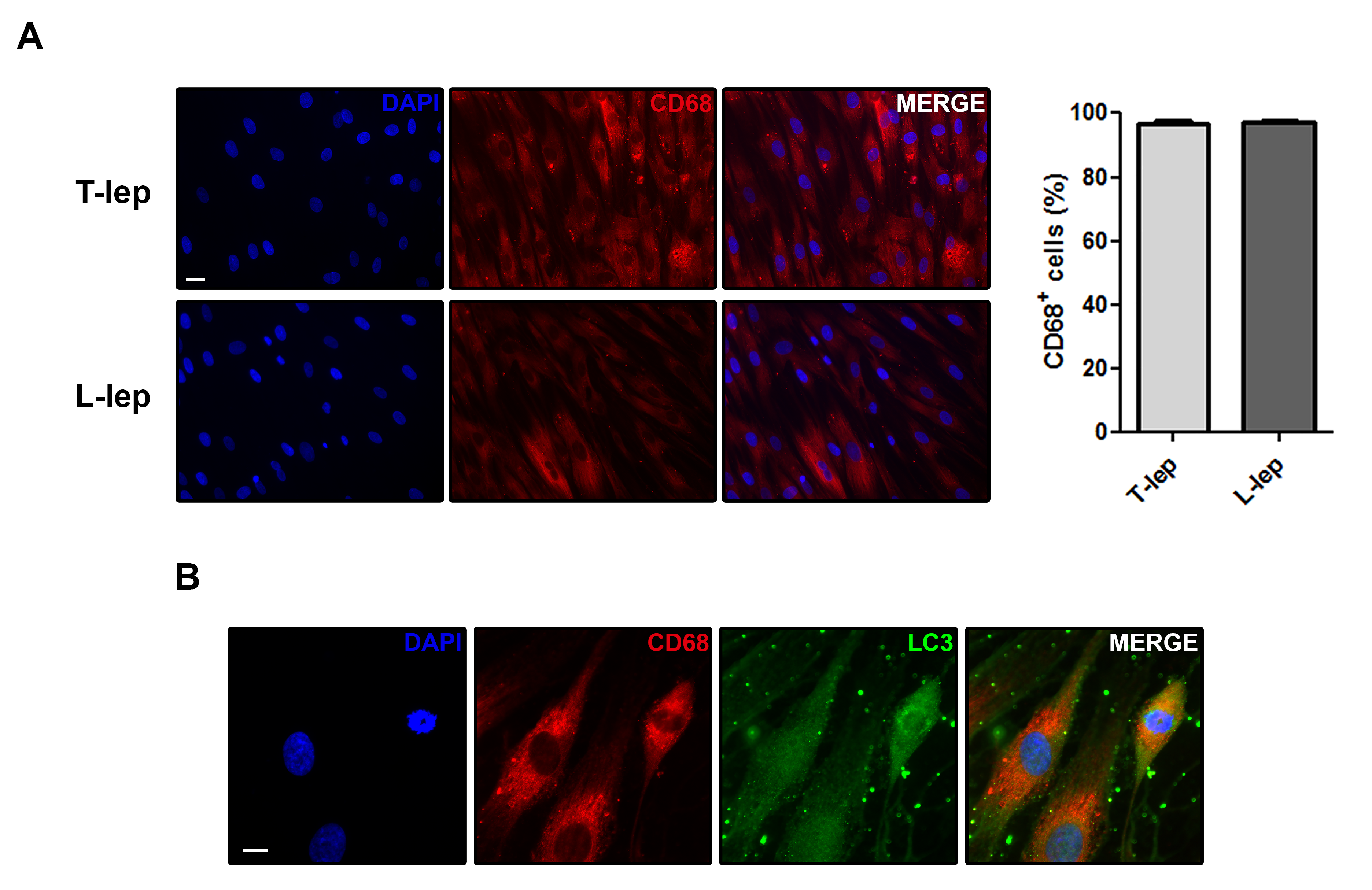

Supplement: S1 Fig — (A and B) Macrophages (MΦs) were isolated from skin lesions of tuberculoid (T-lep) and lepromatous (L-lep) patients and cultivated for 7 days in full medium. Cells were fixed and stained as indicated. DAPI was used to visualize the nuclei (blue). (A) Skin-derived MΦs of T-lep and L-lep patients did not show any significant differences in CD68 expression (red). The percentages of CD68+ cells in immunofluorescence are shown. Data are presented as mean ± SEM (T-lep, n = 3; L-lep, n = 3). Scale bar: 25 μm. (B) Coexpression of CD68 (red) and LC3 (green) in skin lesion MΦs of leprosy patients. The images are representative of a T-lep patient. Scale bar: 10 μm. (TIF) [file ppat.1006103.s001.tif]

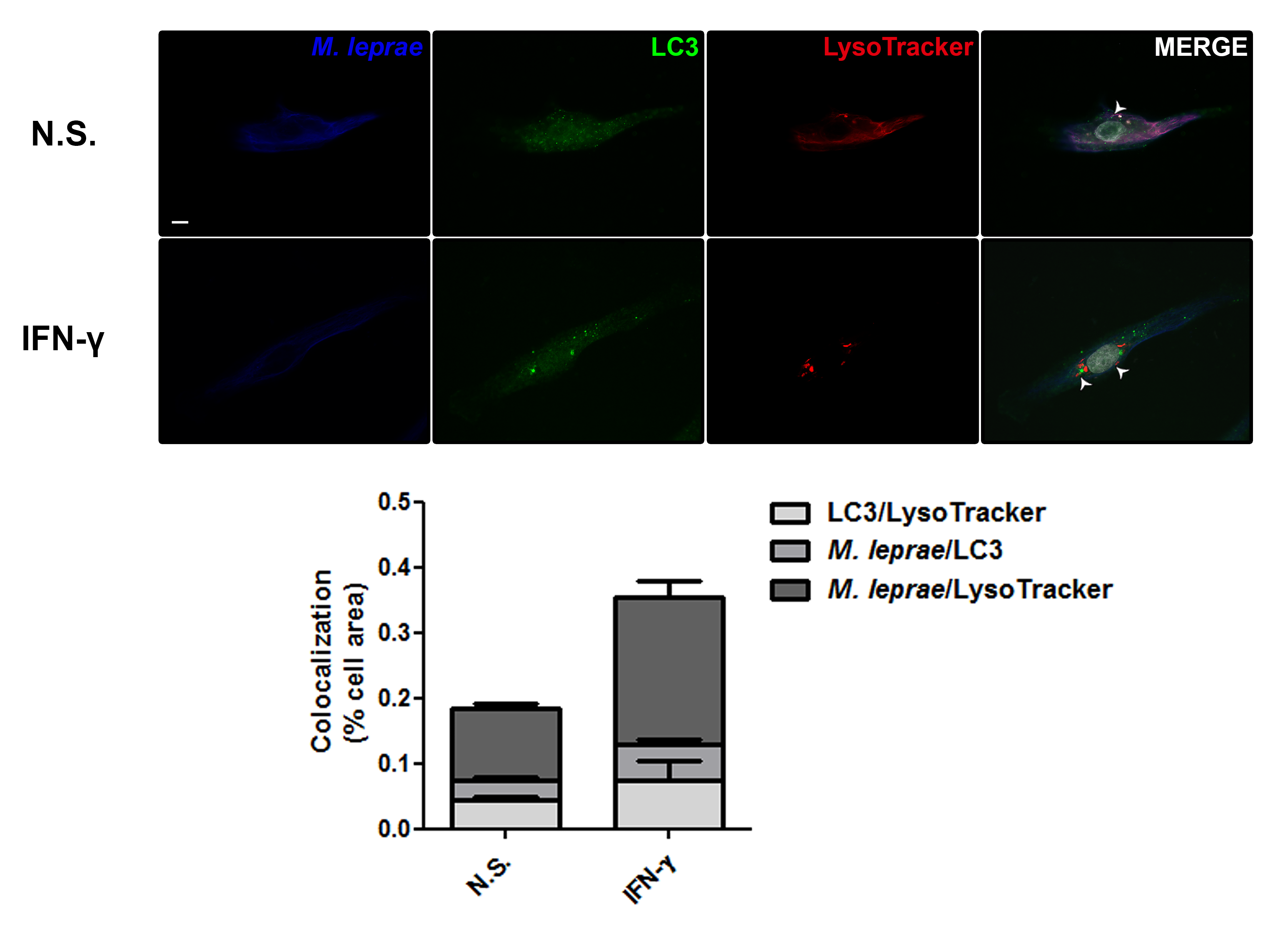

Supplement: S2 Fig — Macrophages (MΦs) were isolated from skin lesions of T-lep patients and incubated in full medium with 10 ng/mL IFN-γ. Eighteen hours after incubation, cells were loaded with 500 nM LysoTracker (red) for 30 min and then fixed and labeled for LC3 (green), M. leprae LAM (blue) and DAPI (white). Fusion profiles between LysoTracker-labeled lysosomes and M. leprae-containing LC3-positive autophagosomes were observed in both non-stimulated (N.S.) and IFN-γ-treated MΦs of T-lep patients. Arrowheads, indicate three-channel colocalization profiles. The images are linked to the experiments described in Fig 4B. Colocalization analysis of immunofluorescence images was performed as indicated and expressed as percentage of cell area. Results represent the mean ± SEM of three independent experiments. Scale bar: 20 μm. (TIF) [file ppat.1006103.s002.tif]

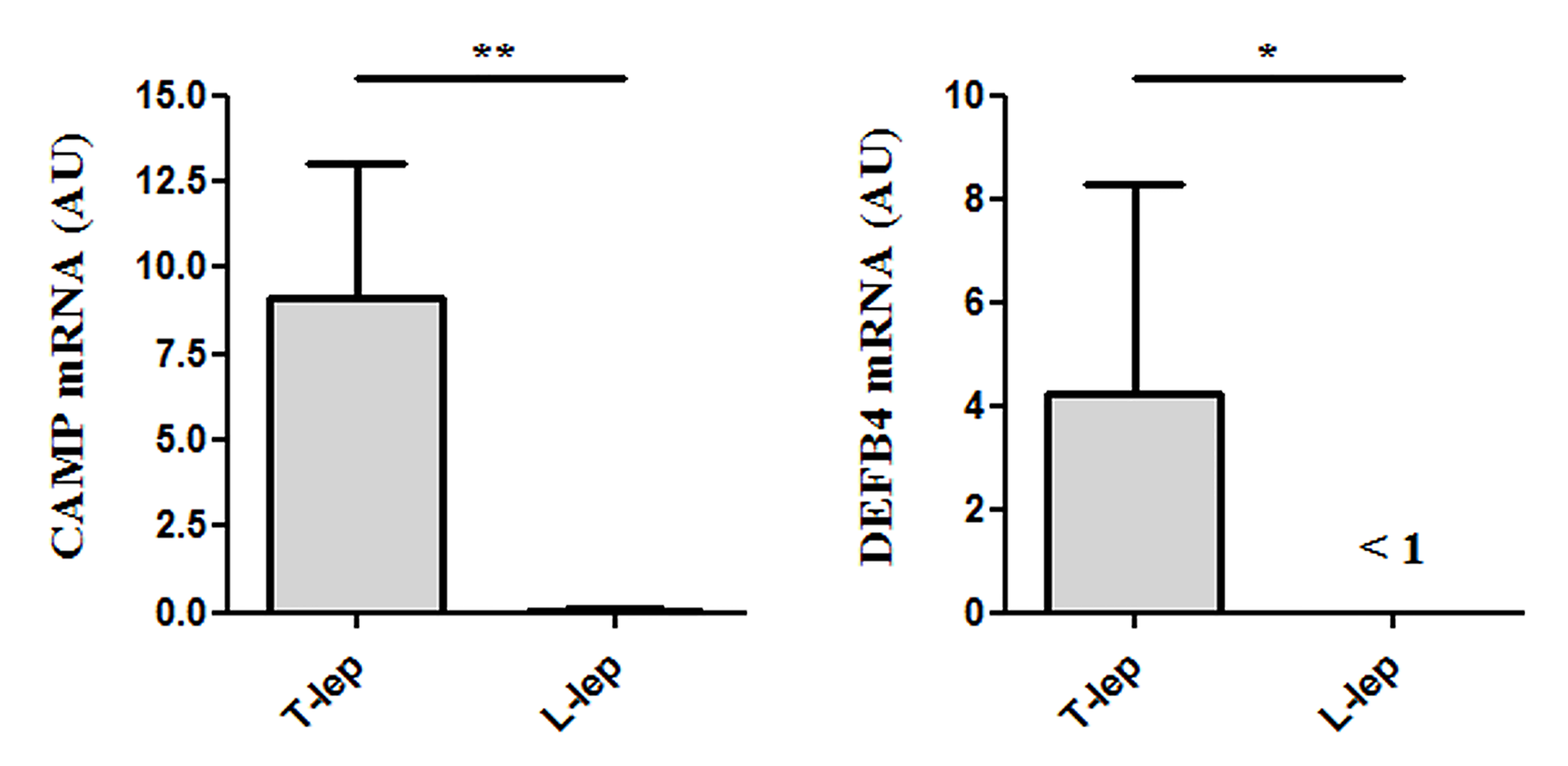

Supplement: S3 Fig — Purified mRNAs from skin lesions of tuberculoid (T-lep) and lepromatous (L-lep) patients were analyzed for cathelicidin (CAMP) and β-defensin 2 (DEFB4A) expression by RT-qPCR. Gene expression data were normalized to GAPDH and mRNA levels are expressed as arbitrary units (AU). Bars represent the mean values ± SEM of six independent samples of each patient group. *P < 0.05, **P < 0.01, Mann-Whitney test. (TIF) [file ppat.1006103.s003.tif]

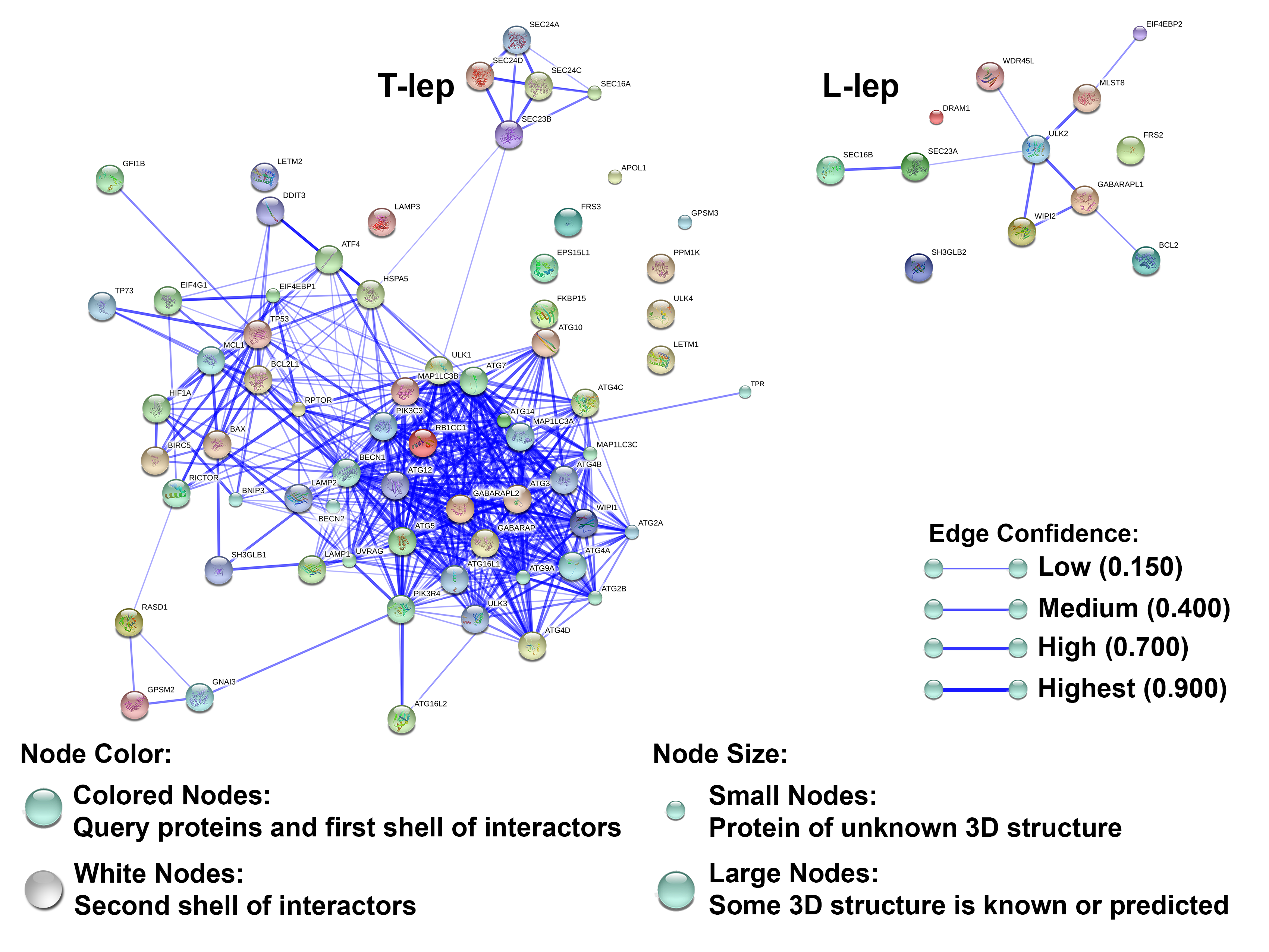

Supplement: S4 Fig — Genes with a differential expression in leprosy lesions by autophagy PCR array analysis were visualized by STRING. The confidence network view. In this view, the color thickness of the edges represents the confidence score of a functional association. Network nodes represent genes. Edges represent gene-gene associations. Interactions among autophagy-associated genes were more predominant in tuberculoid (T-lep) than lepromatous (L-lep) patients. Gene networks are linked to the experiments described in Fig 5. Interaction maps are representative of four T-lep and seven L-lep samples. (TIF) [file ppat.1006103.s004.tif]

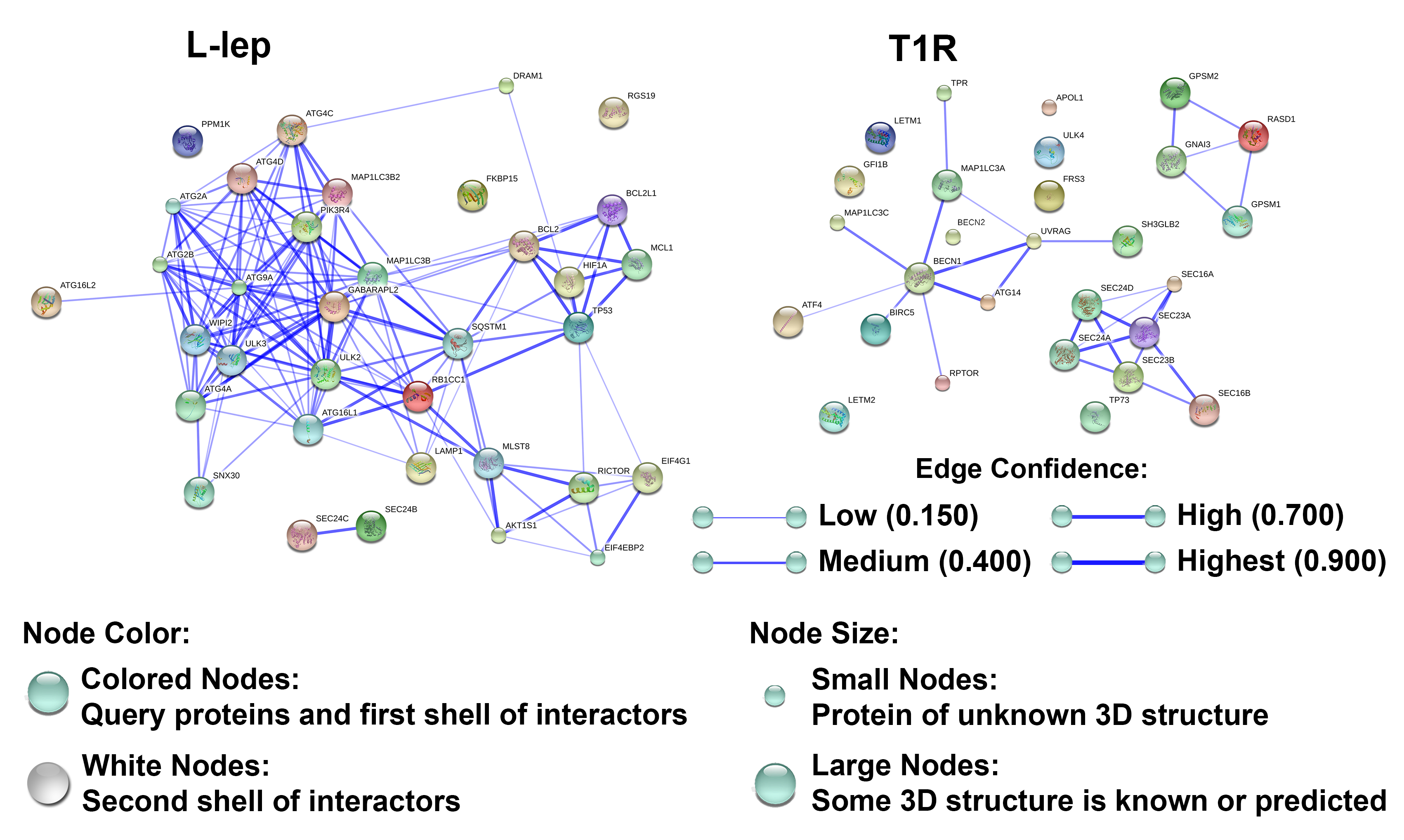

Supplement: S5 Fig — Genes with a differential expression in leprosy lesions according to autophagy PCR array analysis were visualized by STRING. The confidence network view. In this view, the color thickness of the edges represents the confidence score of a functional association. Network nodes represent genes. Edges represent gene-gene associations. Interactions among autophagy processes-related genes were more evident in lepromatous (L-lep) than type 1 reaction (T1R) patients. Gene networks are linked to the experiments described in Fig 7. Interaction maps are representative of seven L-lep and seven T1R samples. (TIF) [file ppat.1006103.s005.tif]
